# Targeting RNA-binding proteins with small molecules: perspectives and challenges

**DOI:** 10.3389/fchem.2025.1649692

**Published:** 2025-10-02

**Authors:** Olivier Mpungi Konde, Williams Balela Balela, Tania Bishola Tshitenge

**Affiliations:** 1 Department of Life Sciences, Faculty of Sciences, University of Kinshasa, Kinshasa, Democratic Republic of Congo; 2 One Health Institute for Africa (INOHA), University of Kinshasa, Kinshasa, Democratic Republic of Congo; 3 Department of Epidemiology and Global Health, Institut National de Recherche Biomédicales (INRB), Kinshasa, Democratic Republic of Congo

**Keywords:** drug discovery, RNA-binding proteins, small molecules, RNA-protein interactions, PROTACs, natural products, molecular glues

## Abstract

RNA-binding proteins (RBPs) play critical roles in numerous biological processes because they regulate RNA function by directly interacting with RNA molecules. In recent years, researchers have developed small molecules that can affect the function of RBPs, opening up promising new directions for drug discovery. While several reviews have already explored this topic, here we aim to provide additional perspectives and highlight emerging challenges in the area of targeting RBPs. There are several types of small molecule modulators that are particularly developing in this field. These include molecules that bind directly to RBPs and alter their interaction with RNA, bifunctional molecules that associate to either RNA or RBPs to disrupt or enhance their interaction, and other compounds that affect the stability of either the RNA or the RBP itself. Among these, bifunctional molecules stand out as especially promising, as they offer potential solutions to some of the common challenges faced in developing drugs targeting RBPs.

## 1 Introduction

In eukaryotic cells, gene expression is tightly regulated through several key processes, including transcription, pre-messenger RNA (pre-mRNA) splicing, mRNA polyadenylation and mRNA editing (Clayton, 2013; Romano and Buratti, 2013). The regulation of these RNA-related processes depends on a variety of molecular interactions, particularly those involving proteins that bind RNA or interact with other proteins (Castello et al., 2016; Gerstberger et al., 2014; Nguyen et al., 2018; Sternburg and Karginov, 2020). Among these, RNA-binding proteins (RBPs) are crucial regulators, with around 2,000 RBPs identified in humans, making up roughly 7.5% of the human proteome.

These proteins interact with both coding mRNAs and noncoding RNAs, such as microRNAs, small interfering RNAs (siRNAs), and small nuclear RNAs (snRNAs), to control RNA function (Clayton, 2013; Qin et al., 2020; Wang S. et al., 2022).

RBPs are grouped into various families based on their structure and function. For example, the Hu antigen R (HuR) family regulates a broad range of transcripts (Wu and Xu, 2022), while the heterogeneous nuclear ribonucleoproteins (hnRNPs) are essential for nucleic acid metabolism (Geuens et al., 2016). Other important families include the arginine/serine-rich splicing factors, which may be localized in the nucleus or shuttle between cellular compartments (Zheng et al., 2020), and the RNA-binding motif (RBM) proteins, which are involved in different types of cancer (Qin et al., 2020; Salicioni et al., 2000; Wu and Xu, 2022). Despite their diversity, all RBPs have at least one RNA-binding motif that allows them to interact with specific RNA sequences or structures (Bheemireddy et al., 2022). These interactions are critical for multiple aspects of RNA biology, such as splicing, polyadenylation, stability, transport, and translation (Wang S. et al., 2022). Because RBPs play central roles in these processes, they are increasingly recognized as important in disease development and as promising targets for drug discovery (Li and Kang, 2023). RBPs may also serve as biomarkers for various clinical applications. Structurally, RNA-binding motifs are conserved protein domains that recognize specific RNA sequences, motifs, secondary structures, or chemical modifications. These RNA–RBP interactions are highly specific and essential for accurate post-transcriptional gene regulation (Clayton, 2013; Clayton, 2019; Li Z. et al., 2021).

RBP-targeting strategies are entering a new era. As our understanding of RNA-binding protein (RBP) biology deepens and innovative drug-discovery modalities emerge, the first successful cases of RBP modulation have become prominent. A typical example is Nusinersen (Spinraza), an antisense oligonucleotide (ASO) that modulates splicing of SMN2 to compensate for loss of SMN1 in spinal muscular atrophy (SMA). This disease is caused by inactivated mutations in the SMN1 gene; the nearly identical SMN2 gene produces only ∼10% of full-length SMN due to predominant exclusion (>90%) of exon 7 in the mature transcript. Nusinersen restores exon inclusion, increasing SMN protein levels and dramatically improving clinical outcomes (Qiu et al., 2022). Nusinersen binds the Intronic Splicing Silencer N1 (ISS-N1) within intron 7 of SMN2 pre-mRNA. It displaces hnRNP proteins at that silencer site, promoting exon 7 inclusion and increasing full-length SMN protein production. Clinical trials such as ENDEAR and CHERISH demonstrated that Nusinersen significantly improves motor function and survival compared to controls, leading to regulatory approval in multiple regions including the USA and EU (Zanoteli et al., 2024).

Research into RBP-targeted therapeutics for cancer is rapidly advancing, and several promising candidates have reached the clinical trial stage. Approaches in development include small-molecule inhibitors (SMIs), ASOs, aptamers, peptides, and molecular glues. Notably, RBPs such as eIF4F, FTO, SF3B1, nucleolin, and RBM39 are under active investigation, some with agents already in early-phase clinical trials (Jungfleisch and Gebauer, 2025). Beyond these, PRMT5 stands out as the only other well-documented RBP now being clinically evaluated for oncology indications. PRMT5 inhibitors like GSK3326595 (also known as EPZ015938), JNJ-64619178 (Onametostat), PF-06939999, PRT543, PRT811 and SCR-6920 are either in Phase I or Phase II trials for various cancers, including solid tumors and hematologic malignancies, often characterized by spliceosome mutations or MTAP deletions. For example, PRT543 has shown early signals of activity in adenoid cystic carcinoma and myeloid malignancies, with pharmacodynamic reductions in symmetric dimethylarginine and manageable safety profiles (Araki et al., 2023).

Recent advances in structural biology, including techniques like X-ray crystallography and nuclear magnetic resonance (NMR) spectroscopy, have provided detailed insights into how RBPs recognize RNA (Araki et al., 2023; Barnwal et al., 2017; Corley et al., 2020; Jungfleisch and Gebauer, 2025; Muppirala et al., 2011; Qiu et al., 2022; Rhodes et al., 2024; Zanoteli et al., 2024). Computational methods, such as deep learning models and RNA–protein interface databases, are being used to predict RNA–RBP interactions (Zhang and Ferré-D’Amar, 2014; Cantara et al., 2017). Deep convolutional and recurrent neural networks have also emerged as powerful tools for identifying RNA-binding proteins (Tants and Schlundt, 2023; Thompson et al., 2019). However, these predictions still rely on experimental validation. Because RNA–RBP interactions can be transient or stable, they offer regulatory points across various stages of an RNA molecule’s life, from its generation and maturation to its degradation. Disruptions in the interactions between RNAs and RBPs have been linked to diseases, including cancer, highlighting the therapeutic potential of targeting RBPs (Nagasawa et al., 2024; Qin et al., 2020; Wang S. et al., 2022).

Despite being considered “undruggable” due to the absence of classic binding pockets, some RBPs have been successfully targeted by small molecules (Julio and Backus, 2021). These small molecule inhibitors work through various mechanisms to modulate RNA–RBP interactions and demonstrate potential for therapeutic use (Fang et al., 2024; Pan et al., 2018). In this review, we explore the structural motifs that allow RBPs to bind RNA, summarize the mechanisms by which small molecule inhibitors affect RBP function, and outline strategies for developing such molecules. Collectively, recent research supports the feasibility of designing small molecules to regulate RBPs, offering a new path forward for treating diseases influenced by RNA–RBP interactions.

## 2 RNA-binding domains: key modules of RNA-binding proteins

RNA-binding proteins (RBPs) play a vital and widespread role in regulating RNA transcripts throughout their entire life cycle (Corley et al., 2020). They interact with RNAs in various ways, ranging from simple interactions involving a single RBP and RNA element to the formation of large complexes like the spliceosome, which involve multiple proteins and RNA molecules. Although much progress has been made in identifying these interactions, the precise mechanisms by which RBPs selectively bind their RNA targets remain only partially understood.

RBPs contain specialized RNA-binding domains (RBDs), which are the main functional units responsible for binding RNA. Many RBPs possess multiple RBDs, enabling them to bind RNA more effectively through cooperative or modular interactions (Pereira et al., 2017; Zhao et al., 2023). Additionally, RBPs are often rich in intrinsically disordered regions (IDRs), which can also bind RNA, though their lack of stable structure limits structural studies to more ordered parts of the proteins (Järvelin et al., 2016). While several RBDs have been studied extensively, the diversity and complexity of these domains make classification difficult (Gerstberger et al., 2014) and many RBPs still lack well-characterized RBDs (Castello et al., 2016).

**FIGURE 1:**
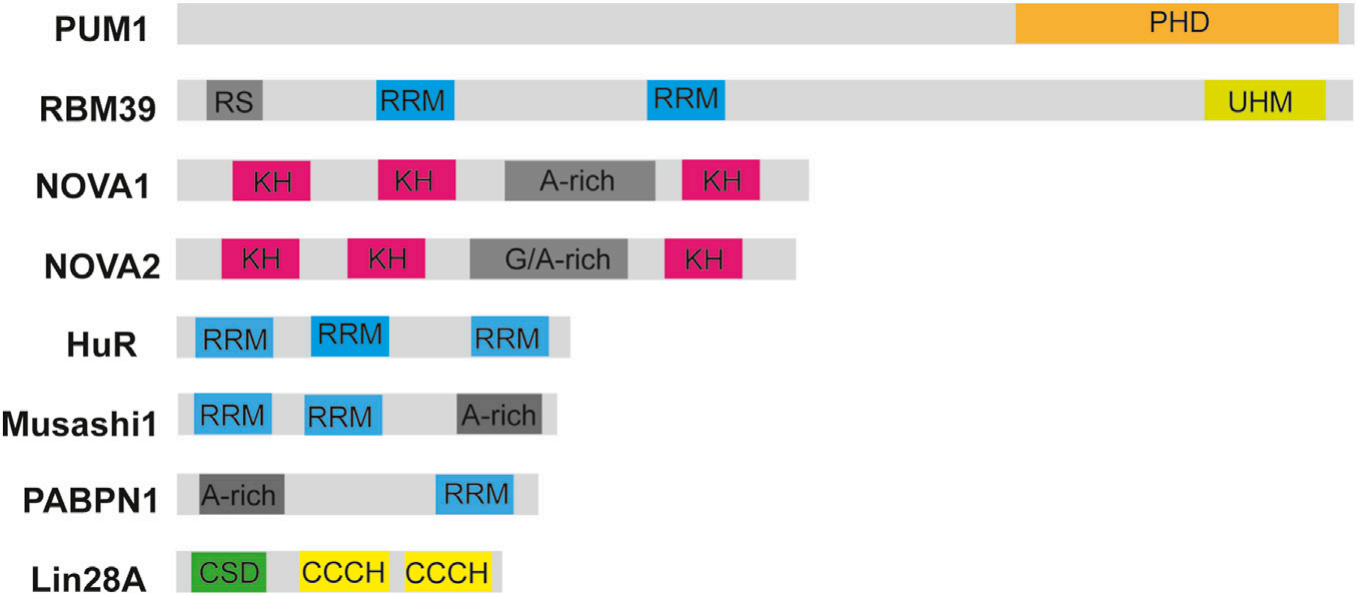
Schematic diagrams of RNA-binding domains in selected RBPs. Diagrams of some RBPs such as the RNA-binding motif protein 39 (RBM39), Musashi-1, Hu antigen R, Neuro-oncological ventral antigen 1 and 2 (NOVA1 and NOVA2) are shown. The RNA-binding motifs are highlighted in different colors such as PHD, pumilio homology domain; CSD, cold-shock domain; RRM, RNA-recognition motif; KH, K homology domain; UHM, U2AF homology motif; CCCH, zinc finger domain. RS is a region rich in arginine and serine residues. Figure was made in Adobe illustrator by TBT.

Some of the most well-known RBDs, identified through structural techniques like NMR and X-ray crystallography, as illustrated in Figure 1, include the RNA recognition motif (RRM), which is found in approximately 0.5%–1% of human genes, as well as the K homology (KH) domain, double-stranded RNA-binding domain (dsRBD), cold-shock domain (CSD), zinc fingers (ZnF), pumilio homology domain (PHD), and intrinsically disordered regions such as the arginine-glycine–glycine (RGG) motif and tyrosine-rich regions. These domains, along with other less common ones, are detailed in Table 1. Additionally, RNA helicases also bind RNA and are essential for its regulation, with several small-molecule inhibitors developed against viral helicases (Li and Kang, 2023; Zhang and Ferré-D’Amar, 2014).

**TABLE 1:** RNA-binding motifs in RBPs.

Motifs	Characteristics	References
RRM (RNA Recognition Motif)	Also known as RBD (RNA-binding domain) or RNP (ribonucleoprotein domain). It is composed of 75–85 amino acids and has a structure of a beta-sheet with four strands and two alpha-helices. It is the most abundant RNA-binding domain in RBPs	Li et al. (2021a), Maris et al. (2005)
KH Domain	This domain contains about 70 amino acids and is found across many organisms. In eukaryotes, it usually contains a beta-sheet with three antiparallel strands and three alpha-helices	Grishin (2001), Olejniczak et al. (2022), Valverde et al. (2008)
dsRBD (Double-Stranded RNA-Binding Domain)	This domain binds to double-stranded RNA and also interacts with proteins. It has around 70 amino acids with a structure made of three strands and two helices	Masliah et al. (2013), Tian et al. (2004)
CSD (Cold Shock Domain)	It is made of ∼70 amino acids that form a five-stranded beta-barrel structure. It binds single-stranded RNA or DNA and often works with flexible/unstructured protein regions	Heinemann and Roske (2021), Lindquist and Mertens (2018), Mayr et al. (2012)
ZnF (Zinc Finger)	Typically binds DNA, but some (like CCCH-type) bind RNA. Around 60 human proteins of this type are known to bind RNA. These proteins often shuttle between cell compartments and have other domains for added functions	Bishola and Clayton (2022), Cassandri et al. (2017), Sievers et al. (2018)
RGG Motif	Contains the sequence RGG, which is evolutionarily conserved. It binds G-quadruplex structures in RNA and is also involved in protein interactions	De Vries et al. (2022), Masuzawa and Oyoshi (2020), Vasilyev et al. (2015)
PUM-HD (Pumilio Homology Domain)	The domain is made of eight repeated units (each 36 amino acids). It forms PUM repeats that recognize RNA sequences	Qiu et al. (2019), Wang et al. (2001), Wang et al. (2018a)
Other Motifs	They include domains like leucine-rich repeats (e.g., in Toll-like receptors). They also include unstructured regions (like IDRs), which are rich in amino acids like tyrosine and phenylalanine and can form fibers or hydrogels to interact with RNA.	Dalpke and Helm (2012), Rhodes et al. (2024), Zigdon et al. (2024)

Interestingly, not all RBPs contain known RNA-binding motifs. Some non-traditional RBPs still associate with RNA and form complex assemblies (Castello et al., 2016). These RBP–RNA complexes often exhibit intricate structures involving folded and unfolded regions, and multiple types of intermolecular interactions (Lovci et al., 2016). Domains beyond the canonical RBDs can also contribute to the stability and function of these complexes. Thus, further structural studies of ribosomes, spliceosomes, tRNA synthetases, and RNA polymerase complexes are crucial for deepening our understanding of RBP–RNA interactions (He et al., 2023).

Structural biology has proven to be a powerful approach for uncovering the molecular basis of these interactions. High-resolution structures derived from cryo-electron microscopy (cryo-EM), X-ray crystallography, and NMR have been deposited in the Protein Data Bank (PDB). These structures provide detailed insights into molecular interactions, but determining RBP–RNA complex structures remains challenging (Antoine et al., 2012; Lunde et al., 2007). Several factors contribute to this difficulty: RBPs often have multiple domains connected by flexible regions, making them dynamic and harder to crystallize; RNA recognition can involve transient interactions; and RNA molecules themselves are highly dynamic and may undergo structural changes upon protein binding (Järvelin et al., 2016; Lunde et al., 2007). Because of these challenges, integrating multiple experimental approaches is often necessary. For example, NMR is excellent for studying protein and RNA dynamics, but it is limited by the size of the complexes. Combining NMR with techniques like electron paramagnetic resonance (EPR), small-angle X-ray scattering (SAXS), X-ray crystallography, and cryo-EM can yield a more comprehensive view of RBP–RNA structure and dynamics (He et al., 2023; Lovci et al., 2016).

Recent breakthroughs in AI-driven protein structure prediction have markedly accelerated structural biology and opened new avenues in understanding biomolecular interactions. Since its launch, AlphaFold2 has enabled the accurate modeling of complex protein structures, including larger protein assemblies for which only sparse or low-resolution experimental data were available. This transformative tool has empowered researchers to generate high-confidence structural hypotheses, which can now be experimentally validated far more efficiently than in the pre-AlphaFold2 era. In May 2024, AlphaFold3 was introduced, marking a significant leap forward. Unlike its predecessor, AlphaFold3 is designed to predict not only protein structures but also RNA secondary structures and protein–RNA complexes capabilities previously unavailable in AlphaFold2. This innovation holds particular promise for the RNA-binding protein (RBP) field, as it allows researchers to model dynamic RNA–protein interfaces critical to understanding post-transcriptional regulation and to design inhibitors targeting these interfaces. The ability to predict protein–RNA interactions *in silico* offers an unprecedented opportunity to accelerate drug discovery against RBPs once considered structurally elusive (Varadi et al., 2024).

As described in Kilim et al., 2023, the structure of the SARS-CoV-2 spike protein RBD (amino acids 331–531, based on NCBI RefSeq: YP_009724390.1, UniProt ID: P0DTC2) was predicted using AF2. The predicted structures were aligned with the RBD region of the experimentally resolved RBD–ACE2 complex (PDB ID: 6M0J, Chain B [auth E], residues 15–208) using all-atom alignment to minimize the Root Mean Square Deviation (RMSD) between the structures (Kilim et al., 2023). AF2 predictions provided valuable insights into both monomeric and multimeric vaccine formulations, revealing that monomers expose more antigenic epitopes. This structural clarity was particularly crucial for evaluating potential mutations, such as those in the Omicron BA.1 variant that enhance ACE2 binding affinity and allow immune escape. Notably, AF2’s accuracy has reached the level where it can assist in interpreting X-ray crystallographic diffraction data and guide experimental model building (Ali and Caetano-Anollés, 2024; Gutnik et al., 2023). However, limitations remain. The AF2 algorithm tends to favor thermodynamically stable conformations, in part due to biases in its training data, and it relies on multiple sequence alignments (MSAs) that reflect ground-state structures. Consequently, it may struggle to accurately predict alternative or transient protein conformations, especially in systems where conformational heterogeneity plays a functional role (Raisinghani et al., 2024). To address these limitations, single-molecule fluorescence techniques particularly smFRET (single-molecule Förster Resonance Energy Transfer) have been used to study conformational dynamics and protein–RNA interactions in real time. For example, smFRET was applied to investigate allosteric regulation of the SARS-CoV-2 spike protein, showing that ligand binding modulates the equilibrium of RBD conformations and thereby affects receptor exposure and antibody accessibility (Agam et al., 2023; Raisinghani et al., 2024).

Mutations disrupting RBD structure or function are increasingly recognized as causative in human diseases, particularly in neurodegenerative disorders. Mutations in TDP-43 and FUS/TLS, both RBPs implicated in amyotrophic lateral sclerosis (ALS) and frontotemporal dementia (FTD) alter RBD stability, aggregation propensity and RNA-binding specificity, leading to widespread perturbation of RNA processing, including splicing, transport and decay (Loughlin and Wilce, 2019). For instance, familial ALS-linked mutations in TDP-43 enhance protein instability, promote abnormal associations with FUS, and lead to nuclear clearance and cytoplasmic aggregation; events thoughts to undermine normal metabolism and contribute to neurotoxicity (Ling et al., 2010).

Beyond structural insights, research has shown that dysregulation or malfunction of RBPs plays a critical role in cancer progression. Imbalances in RBP activity can disrupt the post-transcriptional regulation of oncogenes and tumor suppressor genes, leading to hallmarks of cancer such as uncontrolled proliferation and resistance to cell death. For instance, AU-rich element RNA-binding protein 1 (AUF1) stabilizes specific mRNAs by binding to AU-rich elements in the 3′-untranslated regions (UTRs), thereby enhancing the expression of genes involved in cancer cell proliferation and survival (Jungfleisch and Gebauer, 2025; Lei et al., 2025; Wang et al., 2025). Similarly, HuR (ELAVL1) is a ubiquitously expressed RNA-binding protein that recognizes adenine- and uridine-rich elements (AREs) within the 3′untranslated regions (UTRs) of target mRNAs. Through this binding, HuR regulates mRNA stability and translation, influencing key cellular processes such as proliferation, survival, angiogenesis, invasion, and metastasis. HuR is frequently overexpressed in diverse cancer types and has emerged as a critical player in tumor progression by stabilizing oncogenic mRNAs (Brennan and Steitz, 2001; Soomro et al., 2020). Structurally, HuR consists of three RNA recognition motifs (RRMs): two N-terminal RRMs responsible for ARE binding, connected via a basic hinge region to a third C-terminal RRM. Understanding the molecular interactions between HuR and small-molecule inhibitors is essential for delineating their therapeutic potential and minimizing off-target effects (Brennan and Steitz, 2001).

## 3 Small molecules targeting RNA-binding proteins and mRNA splicing

RNA-binding proteins (RBPs) are essential regulators of gene expression and are increasingly targeted in drug discovery due to their role in various diseases, such as neurodegenerative disorders, cardiovascular diseases, and cancers (Li and Kang, 2023). Dysregulation of RBPs, such as altered interactions with RNA, phase separation, or aberrant expression, contributes to these diseases (Wheeler et al., 2024). RBPs contain RNA-binding domains (RBDs), including the RNA recognition motif (RRM), K homology (KH), DEAD/DEAH helicase, and zinc finger domains. These domains enable RBPs to recognize specific RNA sequences and structural motifs, providing opportunities for therapeutic targeting (Jungfleisch and Gebauer, 2025; Schmeing and Hart, 2024). We will discuss here progress that have been made in targeting RBPs as well as RNA-RBD interactions in regard to several diseases.

Antisense oligonucleotides (ASOs), such as Nusinersen, have emerged as a strategy to block RBP-RNA interactions in diseases like spinal muscular atrophy (SMA). Nusinersen, for instance, works by hybridizing with RNA in a sequence-specific manner, thereby modifying splicing events (Zanoteli et al., 2024). According to Singh et al. (2006), the stimulation of exon 7 inclusion by antisense oligonucleotides is due solely to the blocking of the intronic splicing silencer N1 (ISS-N1) element. As deregulated splicing has been implicated in numerous cancers and neurodevelopmental disorders, components of the spliceosome, like SF3B1, are potential drug targets. Spliceostatin, a methylated pladienolide derivative of FR901464 and E7107 as well as sudemycins have shown antitumor activity by modulating splicing processes (Kaida et al., 2007; Shi et al., 2020; D’Agostino et al., 2019). These compounds inhibit the binding of U2 snRNA to pre-mRNA, preventing the spliceosome from transitioning to the catalytically active form. A small molecule modulator, H3B-8800, derived from E7107, has shown effectiveness in blocking splicing by inhibiting the SF3B1 complex, particularly in mutant hematological tumor cells. Molecular dynamics simulations indicate that both H3B-8800 binding and the common K700E mutation in SF3B1 influence the internal motion and conformational dynamics of the SF3b complex, which may in turn affect interactions with other spliceosome subunits and global spliceosome stability (Spinello et al., 2021). Bulk RNA-sequencing in SF3B1-mutant (e.g., K700E) cancer cells treated with H3B-8800 reveals preferential retention of short, GC-rich introns, especially those under ∼300 nt in length. Many of the affected transcripts encode core spliceosome components, establishing a feedback loop where splicing modulation leads to impaired expression of spliceosomal machinery, enhancing specificity for mutant SF3B1 cancer cells (Seiler et al., 2018). RNA-sequencing from peripheral blood samples of patients treated in early-phase clinical trials of H3B-8800 shows a dose-dependent increase in alternative splicing events, predominantly exon skipping and intron retention. In high-dose patients, a significant proportion of DNA repair genes exhibit mis-splicing, including BRCA1 exon skipping, correlating with downregulation of expression and impaired DNA repair capacity (Wheeler et al., 2024).

Small molecules targeting RNA offer a new avenue for modulating RBP interactions (Li and Kang, 2023). These small molecules along with their mechanisms of actions and methods of identification are detailed in Table 2. One prominent example is the regulation of SMN2 exon 7 splicing, an attractive target for spinal muscular atrophy (Sivaramakrishnan et al., 2017). Compounds like NVS-SM1 and NVS-SM2, which enhance U1 snRNP binding to SMN2 pre-mRNA, improve exon 7 inclusion, leading to higher levels of functional SMN protein (Palacino et al., 2015). Other small molecules, such as risdiplam and branaplam, have been tested in clinical trials for spinal muscular atrophy, all aiming to promote splicing and increase SMN protein levels.

**TABLE 2:** Small molecules targeting RBPs, their mechanisms of action and methods of identification.

Compounds	Mechanisms of action	Methods of identification	References
Ribosome-targeting antibiotics	Inhibit translation by binding ribosomal RNA	Various established biochemical and structural methods	Lin et al. (2018)
Spliceosome SMN-C1, -C2, -C3, -C5, NVS-SM1, NVS-SMN2	Enhance binding of U1 snRNP to the 5′splice site of SMN2 exon	High-throughput screening (HTS), firefly luciferase assay	Naryshkin et al. (2014)
RG7800, Risdiplam	Modify alternative splicing of SMN2 pre-mRNA	HTS, luciferase reporter, lead optimization	Ratni et al. (2016)
Branaplam	Stabilizes interaction between spliceosome and SMN2 pre-mRNA	HTS phenotypic screen, lead optimization	Cheung et al. (2018)
Spiro[indol-3′,2-pyrrolidin]-2(1H)-one	Inhibits the helicase activity of Brr2, affecting spliceosome dynamics	HTS ATPase assay, lead optimization	Gollner et al. (2016)
Topotecan	Blocks U4 RNA–NHP2L1 interaction	Time-resolved FRET assay	Lambrecht et al. (2024)
SF3B complex (e.g., Pladienolide B, E7107, H3B-8800)	Competitively blocks pre-mRNA binding by stalling the SF3B complex in open conformation	Activity-based protein profiling (ABPP), medicinal chemistry	Kotake et al. (2007)
FR901464, Spliceostatin A	Inhibit splicing machinery and induce cell cycle arrest; derived from natural products	Natural product screening	Kaida et al. (2007)
Sudemycins	Synthetic analogs of spliceostatin A with similar splicing inhibitory effects	Rational design, medicinal chemistry	Lagisetti et al. (2008)
Sudemycinol C and E, Milciclib, PF-3758309, PF-562271	Inhibit splicing and splicing factor kinases	TESLR assay (Triple Exon Skipping Luciferase Reporter)	Owa et al. (1999)
RBM39 (Indisulam, E7820)	Promote RBM39 degradation by recruiting it to an E3 ubiquitin ligase	Forward genetics and phenotypic screening	Han et al. (2017)
HuR Dehydromutacin, MS 444 and okicenone	Inhibit HuR dimerization, blocking RNA-binding function	HTS using confocal fluctuation spectroscopy assay	Meisner et al. (2007)
KH-3	Disrupts HuR–FOXQ1 RNA interaction	HTS fluorescence polarization (FP) assay	Wu et al. (2020)
EIF4E-Arylsulfonyl fluoride	Irreversibly inactivates EIF4E via covalent labeling at Lys162	Covalent docking and structure-based design	Wan et al. (2020)

Similarly, milciclib, PF-3758309, and PF-562271 have been identified as potent splicing modulators in antitumor applications (Ratni et al., 2018; Shi et al., 2020; Singh et al., 2020). Among HuR-targeting compounds, MS-444 has demonstrated nanomolar affinity and specificity, effectively disrupting the HuR–RNA interaction. Crystallographic and NMR data show that MS-444 binds to a hydrophobic cleft between RRM1 and RRM2, interfering with RNA docking and promoting cytoplasmic retention of HuR. This structural disruption impairs HuR’s function in stabilizing pro-tumorigenic transcripts, contributing to its anti-proliferative, anti-angiogenic, and anti-inflammatory effects (Blanco et al., 2016; Wang et al., 2019). Importantly, preclinical studies report favorable pharmacokinetics and low toxicity of MS-444 in mice, suggesting that inhibition of HuR does not significantly disrupt essential physiological functions in adult tissues. However, caution remains warranted, as HuR plays a fundamental role in many cellular processes. Its inhibition could, under certain conditions, lead to unintended effects, particularly in rapidly regenerating tissues or under stress conditions (Brennan and Steitz, 2001).

Different oxaboroles have been described as potent compounds against *Trypanosoma brucei*. These include the oxaborole 6-carboxamides, the acoziborole (trade name of SCYX-7158 or AN5568), AN7973, AN11736, AN2965 and AN3056 (Waithaka and Clayton, 2022; Wall et al., 2018). AN7973 and acoziborole inhibit mRNA processing, particularly splicing, leading to the accumulation of unspliced and improperly spliced mRNAs. In addition, DNDI-6148 and acoziborole have shown to target specifically the cleavage and polyadenylation specificity factor 3 (CPSF3), affecting therefore mRNA processing and *trans-*splicing (Begolo et al., 2018; Betu Kumeso et al., 2023). DNDI-6148, a benzoxaborole compound, has also emerged as a preclinical candidate for visceral leishmaniasis (Mowbray et al., 2021), demonstrating similar mRNA processing inhibition.

RBPs represent a critical class of proteins for therapeutic targeting due to their diverse roles in gene expression regulation (Julio and Backus, 2021). For example, the Musashi family of RBPs is involved in several cancers. Ro-08-2750, a small molecule inhibitor, has been identified as a competitive inhibitor of Musashi RNA interactions, offering a potential strategy for targeting RRM1-containing RBPs (Walters et al., 2023). Similarly, MS-444, an inhibitor of HuR RNA-binding protein, has shown effectiveness in inducing apoptosis in glioblastoma cells (Blanco et al., 2016; Wang et al., 2019). Other studies have highlighted small molecules that enhance the stability of certain microRNAs, such as enoxacin, which binds TAR RNA-binding protein 2 (TRBP2) and stabilizes tumor-suppressive miRNAs like pre-miR-125a and pre-let-7 (D’Agostino et al., 2019). This approach suggests that modulation of ribonucleoprotein interactions can regulate post-transcriptional gene expression and provide therapeutic benefits. Thiazolo[5,4-e] indolone (C16), identified through small molecule screening, is a PKR inhibitor that has neuroprotective effects by inhibiting cyclin-dependent kinases. Luteolin, a flavonoid, has been shown to disrupt PKR/PACT interactions, inhibiting PKR phosphorylation and affecting inflammation (Fukuda et al., 2024). However, its use in inflammation-related diseases may require caution, as it may also enhance inflammasome activity, suggesting a need for combinatory treatments with other agents like MCC950.

## 4 Other strategies to modulate the functions of RBPs: covalent inhibitors, degraders and natural substances

**FIGURE 2:**
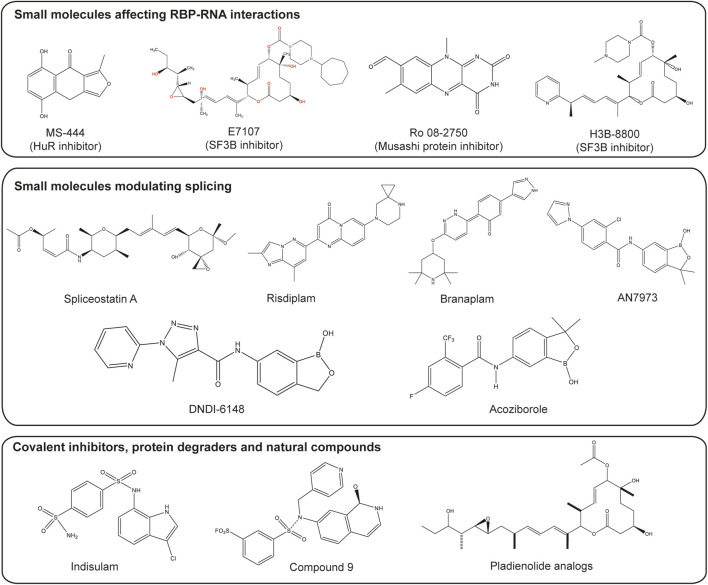
Small molecules targeting splicing and RBPs. Structures of several types of small molecules are shown. These small molecules include those which inhibit RBPs such as MS-444, inhibitor of HuR protein (Meisner et al., 2007), natural-product derivatives E7107 and H3B-8800, which inhibit the splicing factor 3b (SF3b) subcomplex (Seiler et al., 2018; Wheeler et al., 2024) and Ro 08-2750, a competitive inhibitor of Musashi protein (Walters et al., 2023). Small molecules modulating splicing include Risdiplam and Branaplam, modulators of the survival of the motor neuron 2 (SMN2) alternative splicing (Cheung et al., 2018; Ratni et al., 2018); spliceostatin A, a derivative from natural products, which interferes with the binding of U2 snRNA to the pre-mRNA (Yoshimoto et al., 2021); the DNDI-6148 and acoziborole which target the cleavage and polyadenylation specificity factor 3 (CPSF3) (Betu Kumeso et al., 2023; Mowbray et al., 2021). Small molecules with other mechanisms of actions such as covalent inhibitor-compound 9 (Wan et al., 2020), indisulam which induces degradation of RBM39 via recruitment to DCAF15 (Du et al., 2019) and products from natural compounds like pladienolide analogs (Booth et al., 2020) are also included. Figure was made by OMK and WBB in ChemDraw.

Eukaryotic initiation factor 4E (eIF4E) plays an essential role in translation initiation and protein synthesis by binding to the 5′-cap structure of the mRNA (Chen et al., 2012). Recent studies have identified compounds that exhibit potent binding affinity for eIF4E, including compounds 8 and 9. Wan et al. demonstrated that these compounds inhibit eIF4E activity, achieving 68% inhibition with compound 8% and 41% inhibition with compound 9 after treatment with 30 μM for 3 h (Wan et al., 2020). LC-MS/MS analysis revealed that compound 9 primarily modifies lysine 162 (K162) on eIF4E, although K206, located near the m7GTP pocket, was also affected. The co-crystal structure of compound 9 bound to eIF4E (PDB ID 6U09) confirmed that the isoquinolone core of compound 9 interacts with eIF4E similarly to the guanine in m7GTP, confirming its potential as an effective inhibitor (Wan et al., 2020) as shown in Figure 2.

Sumoylation of eIF4E promotes cancer progression by enhancing the formation of an active translation initiation complex, a process dependent on the E3 ligase activity of histone deacetylase 2 (HDAC2). A study by Bayona et al. (2011) demonstrated that *Trypanosoma cruzi* eIF4E is not a target of SUMO modification, despite the presence of other components of the translation initiation complex (Bayona et al., 2011). Identification of eIF4E amino acids covalently bound by SUMO1 was possible by mutating various lysine residues to arginine residues. Five SUMO sites were identified on eIF4E: Lys 36 (IKHP), Lys 49 (FKND), Lys 162 (DKIA), Lys 206 (TKSG) and Lys 212 (TKNR), while a single mutation of these sites did not appear to affect the *in vitro* sumoylation of eIF4E (Xu et al., 2010).

Another promising strategy involves PROTACs (proteolysis-targeting chimeras), which are heterobifunctional molecules designed to induce protein degradation. PROTACs consist of two ligands linked by a linker: one ligand binds to the protein of interest (POI), while the other recruits an E3 ubiquitin ligase. The simultaneous binding of both the POI and ligase induces ubiquitination, leading to the degradation of the target protein via the ubiquitin-proteasome system (UPS) (Schreiber, 2021; Xiao et al., 2022). In most cases, the modified protein is routed to the 26S proteasome, a protease that degrades the substrate into small peptides but allows recycling of the ubiquitin (Li and Hochstrasser, 2000).

In contrast to PROTACs, molecular glues are small molecules that do not contain PROTAC segments but promote the interaction between a target protein and an E3 ligase, leading to protein degradation (Xiao et al., 2022). These molecules induce a conformational change in the target protein, converting it into a “neo-substrate” for the ligase, which then undergoes ubiquitination and degradation (Schreiber, 2021). Despite being discovered serendipitously, molecular glues represent an exciting therapeutic strategy for targeting previously undruggable proteins. For example, indisulam, a sulfonamide used in treating cancers, has been shown to induce the degradation of RBM39 by promoting its interaction with an E3 ligase complex. Further advancements in PROTAC technology have led to the development of a PS-MOE (Phosphorothioate 2′-O-Methoxyethyl) oligonucleotide analog, designed to bind tightly to the zinc finger domain of Lin28A, a key RNA-binding protein. This oligonucleotide was conjugated with a VHL-recruiting peptide, inducing Lin28A degradation via the ubiquitin pathway. This approach represents a novel method for degrading and inhibiting RNA-binding proteins, a class of targets that are typically difficult to treat pharmacologically (Xiao et al., 2022). The details about different types of PROTACs and molecular glues together with their targets and current applications are summarized in Table 3.

**TABLE 3:** Comparison of mechanisms of action, molecular targets and current applications of bifunctional molecules.

Bifunctional molecules	Mechanisms of action	Molecular targets	Current applications	References
PROTACs	They are composed of two ligands: one binds the protein of interest (POI), the other recruits an E3 ubiquitin ligase, leading to ubiquitination and proteasomal degradation	Methionyl aminopeptidase 2 (METAP2); A 3-nitropyridine derivative (N2-(1H-indazole-5-yl)-N6-methyl-3-nitropyridine- 2,6-diamine) named KRIBB11; BRD4 (Bromodomain-containing protein 4)	Targeted protein degradation in cancer, neurodegeneration, and inflammation	Békés et al. (2022), Li et al. (2023), Xue et al. (2020)
RNA-PROTACs	Target RNA-binding proteins by linking RNA-recognition elements to E3 ligase-binding peptides; however, these constructs often suffer from poor stability and membrane permeability	Lin28 (oncogenic RNA-binding protein)	Early-stage applications in cancer therapy, under active investigation	Ghidini et al. (2021), Li et al. (2021b), Modell et al. (2021)
RIBOTACs	Small molecules that recruit RNase L to degrade specific structured RNAs by dimerizing the ribonuclease on the RNA target.	pri-miR-96, pre-miR-21, SARS-CoV-2 RNA genome	Targeting non-coding RNAs in cancer and viral infections (e.g., COVID-19)	Dey and Jaffrey (2019), Haniff et al. (2020)
Molecular Glues	Small molecules that promote or stabilize protein–protein interactions between POI and E3 ligase, leading to degradation; without the need of a linker	Transcription factors, RBM39 (spliceosome factor), IKZF1/3 (cereblon substrates)	Clinically approved agents for myeloma (e.g., lenalidomide), MDS, and refractory hematologic cancers	Li et al. (2024), Sasso et al. (2023), Schreiber (2021)

Natural compounds have historically been important in drug discovery, especially for cancers and infectious diseases. Plants are recognized as a good source of natural compounds, contributing to drug discovery and offering solutions that are potentially more effective than synthetic molecules. For example, natural anticancer compounds like FR901464, herboxiedenes, and pladienolides, isolated from bacteria, target the SF3b subcomplex of the U2 snRNP and disrupt early spliceosome assembly, demonstrating the potential of natural products to modulate RNA splicing processes (Mizui et al., 2004). Natural compounds have also shown promise in treating tropical neglected diseases like trypanosomal infections. Alkaloids and flavonoids found in the aqueous extract of *Adansonia digitata* (baobab fruit) have demonstrated anti-trypanosomal effects against *T. brucei* infections (Ogunleye et al., 2020). This makes baobab fruit pulp a potential candidate for treating human and animal African trypanosomiasis. In addition, pure compounds from *Siphonochilus aethiopicus* (wild ginger) were shown to possess anti-trypanosomal activity. For instance, compounds like 8(17),12E-labdadiene-15,16-dial and sesquiterpenoids exhibited a minimum inhibitory concentration (MIC) of 5.3 µM and 6.9 µM, respectively, against *Trypanosoma brucei*, making them potential alternatives to suramin (MIC 10 µM) (Igoli et al., 2012). This suggests that these compounds could be evaluated for their ability to target RNA-binding proteins in trypanosomes.

Despite their pharmacological potential, natural products face substantial limitations that must be addressed before clinical translation. First, many exhibit poor bioavailability, which can result from low aqueous solubility, rapid metabolic degradation, or inefficient absorption (Daina et al., 2017). Second, the promiscuous binding profiles of many natural compounds raise concerns about off-target toxicity, particularly when administered systemically (Chaudhari et al., 2020; Kiely-Collins et al., 2021). Third, their structural complexity often poses serious challenges in chemical synthesis, structural optimization, and large-scale manufacturing, which complicates drug development pipelines (Newman and Cragg, 2020).

Overcoming these barriers requires multidisciplinary approaches, including the use of semisynthetic derivatives, nanoparticle-based delivery systems, and AI-assisted *de novo* design to improve pharmacokinetics and reduce toxicity. Additionally, mechanistic studies, including chemo-proteomics and structure-activity relationship (SAR) analysis are essential to better understand how these compounds interact with RBPs and other molecular targets (Harvey et al., 2015).

Given the challenges associated with chemical synthesis and the potential toxic effects of natural products, it is essential to carefully design these compounds, understand their mechanism of action and conduct appropriate studies to elucidate their chemical properties. These steps are crucial for the application of these compounds in targeting RNA-binding protein (RBP) function in clinical studies (Li and Kang, 2023).

Both PROTACs and molecular glues (MGs) offer complementary advantages, advancing the therapeutic potential of targeted protein degradation technologies in disease treatment (see Table 3). Molecular glues are small molecules that modulate protein–protein interactions (PPIs) to promote the degradation of proteins of interest (POIs) by facilitating their interaction with E3 ligases. Their small size and structural simplicity contribute to better cellular permeability and potential for oral administration. Notably, they do not exhibit the “hook effect” commonly seen in PROTACs. However, the development of molecular glues remains challenging due to the unpredictable nature of PPIs, with many effective compounds discovered serendipitously. Their identification often depends on innovative screening approaches to pinpoint molecules capable of modulating PPIs effectively (Zhong G. et al., 2024).

In contrast, most conventional PROTACs exceed Lipinski’s Rule of Five due to their high molecular weight (typically 1,000–2,000 Da), which negatively impacts their solubility, permeability, and cellular uptake. This structural complexity also contributes to active transporter-mediated efflux, limiting their overall efficacy. Nonetheless, recent advances in reversible covalent inhibitors provide a promising complement to PROTAC strategies (Li et al., 2023). These inhibitors offer the benefits of prolonged action and improved selectivity, advantages commonly associated with irreversible covalent inhibitors while mitigating the risks of off-target binding and toxicity. This is particularly valuable in the context of diseases that require long-term treatment and high safety margins, such as autoimmune disorders (Kiely-Collins et al., 2021).

Moreover, alternative targeted protein degradation technologies such as hydrophobic tagging (hydrophobic labeling) offer an E3 ligase-independent approach. This method uses a POI ligand conjugated with a highly lipophilic fragment to induce degradation, conferring monospecificity and avoiding the heterospecificity challenges of PROTAC-based strategies (Wang et al., 2023). The integration of artificial intelligence is playing an increasingly important role in accelerating the rational design of PROTACs. For instance, a recent study integrated pharmacokinetic data into PROTAC-DB 3.0, recognizing the importance of druggability in PROTAC development. While these technologies hold promise, they remain in early stages and require robust datasets to reach their full potential (Ge et al., 2025).

Crucially, emerging studies suggest the value of synergistic applications that combine different targeted protein degradation strategies to enhance efficacy. For example, a covalent inhibitor could be used to first alter the conformation of an RNA-binding protein (RBP), thereby increasing the efficiency of its subsequent degradation by a PROTAC. For instance, covalent PROTACs derived from ibrutinib analogs have demonstrated successful degradation of Bruton’s Tyrosine Kinase (BTK) and B Lymphoid Kinase (BLK) in live cells, underscoring the feasibility of linking covalent warheads to PROTAC scaffolds in order to enhance degradation efficiency in otherwise challenging targets (Xue et al., 2020). This layered approach could overcome resistance mechanisms, enhance selectivity, or improve pharmacokinetics.

Similarly, natural products and their derivatives can serve as molecular glue “primers” by modulating protein–protein interactions or post-translational modifications, potentially sensitizing downstream degradation mechanisms. When used in combination with degraders, these molecules can expand the degradable proteome or improve the selectivity and potency of targeted protein degradation approaches. These compounds offer chemical diversity and often possess inherent binding affinity to protein surfaces, qualities which make them attractive starting points for designing degraders that operate synergistically with PROTACs or glues (Kiely-Collins et al., 2021; Li et al., 2023).

Another example involves dual-ligand PROTACs, multi-valent chimeras that incorporatetwo copies each of the POI ligand and E3-ligase ligand. These constructs promote high-avidity, stable ternary complex formation, resulting in enhanced degradation potency (up to tenfold) and prolonged activity *in vitro* and *in vivo*, compared to conventional single-ligand PROTACs. These dual strategies show how multivalent interactions and sequential engagements can synergistically increase degradation efficiency (Chen et al., 2024).

Another emerging area is the dual-function bifunctional molecules, where one “arm” covalently modifies the protein to stabilize a degradation-prone conformation, and the other arm recruits an E3 ligase (functionally similar to a PROTAC), achieving a two-step degradation mechanism with enhanced precision (Huang et al., 2024). By sequentially targeting a protein with two different mechanisms, such as covalent modification followed by PROTAC binding, the degradation rate can be enhanced or steric hindrance overcome. For example, covalent modification of E3 ligases with electrophilic ligands stabilizes PROTAC ternary complexes, thereby increasing degradation kinetics and circumventing the limitations of solely non-covalent binding (Zhou and Xiao, 2020). This strategy can render previously degradation-resistant proteins, whether due to inaccessible binding pockets or resistance-conferring mutations, susceptible when complementary modalities are combined. As evidence, covalent inhibitors of KRAS G12C, paired with PROTAC recruitment, have successfully induced rapid and sustained degradation of mutant KRAS even where direct PROTAC action alone was insufficient (Jia et al., 2024). Moreover, dual-ligand PROTACs (which carry two copies each of the POI ligand and E3 ligase ligand) exhibit up to a tenfold increase in degradation efficiency compared to single-ligand PROTACs, while allowing lower overall dosing, thus reducing off-target activity and maintaining potency (Chen et al., 2024). This exemplifies how synergy between distinct mechanisms can yield greater selectivity and efficacy than either approach alone.

Combining distinct degradation modalities also helps preempt or overcome resistance mechanisms, such as mutations in E3 ligase binding sites or activation of compensatory signaling pathways, a strategy demonstrated in the case of BTK resistance to ibrutinib, where PROTAC degradation bypassed target‐site mutations to restore efficacy (Li et al., 2022; Xue et al., 2020).

Challenges, however, must be carefully navigated when implementing synergistic targeted protein degradation strategies. One major concern is the risk of additive or synergistic toxicity, which may arise from combining multiple active agents or employing complex bifunctional molecules with overlapping or interacting biological effects. Additionally, there can be pharmacokinetic mismatches between agents that differ in bioavailability, half-life, or tissue distribution, complicating dose optimization and timing. From a development standpoint, such combination strategies introduce greater complexity in drug discovery, requiring sophisticated design, synthesis, and optimization processes, as well as more extensive regulatory evaluation. Furthermore, there is the potential for unpredictable biological cross-effects or off-target interactions, especially in cases where the mechanisms of the combined agents intersect within overlapping cellular pathways or interact with unintended proteins. These challenges have been noted in recent studies exploring multi-mechanism degraders and bifunctional agents (Chaudhari et al., 2020; Chen et al., 2024).

## 5 Perspectives

Small molecules are of significant interest in drug discovery due to several advantages such as oral bioavailability. Although RBPs have traditionally been considered “undruggable” targets due to the absence of well-defined binding pockets, recent evidence demonstrates that diverse small molecules can modulate RBP function, highlighting the feasibility of targeting RBPs (as shown in Figure 2). Key aspects for the development of effective RBP regulators include high-throughput screening methods, elucidation of mechanisms of action, and strategic optimization of identified compounds.

### 5.1 Screening technologies to identify small molecules targeting RNA-binding proteins

Cell-based screens have been developed to identify small molecules that modulate RNA splicing. There are a variety of assays available for screening small molecule inhibitors of RNA-binding proteins (RBPs) from various compound libraries. In this section, we discuss some commonly used screening techniques in drug discovery (Jaiswal et al., 2024). Drug discovery, which is often slow and resource-intensive, requires collaboration across multiple disciplines, as success hinges on the right team, the right target, and the right compound. Various screening methods are employed to identify potential drug leads, with high-throughput screening (HTS) being one of the most widely used approaches (Janzen, 2014).

HTS allows the screening of large compound libraries, often numbering in the millions, to find molecules that interact with specific targets, including proteins and protein-protein interactions (PPIs) (Janzen, 2014; Nag et al., 2013). However, HTS is resource-heavy and has a success rate of only around 50% (Macarron et al., 2011).

An alternative to HTS is Fragment-Based Drug Discovery (FBDD), which focuses on smaller libraries, typically containing 1,000 to 15,000 compounds with greater chemical diversity (Wang L. et al., 2022). FBDD has become an essential tool in drug discovery and often relies on techniques such as Nuclear Magnetic Resonance (NMR), Surface Plasmon Resonance (SPR), and X-ray Crystallography to identify compounds that bind to biological targets. While FBDD involves screening fewer compounds, it is highly effective due to the diversity of molecules involved. NMR, in particular, is considered the gold standard for FBDD studies (Wang L. et al., 2022).

The first step to drug discovery is to screen for drug leads from a large pool of compounds. At present, there is a lack of efficient cell-based methods that can rapidly screen compounds by detecting apoptotic cell death. For example, the commonly used *in vitro* caspase activity assay utilizes cell extracts from a large population of cells. Furthermore, although the TdT-mediated dUTP nick end labeling method is only applicable to fixed cells but not living cells, assays involving annexin V require the use of a fluorescent microscope or fluorescence-activated cell sorting analysis. These methods are time-, labor- and cost-consuming and therefore, will be difficult for a high-throughput drug screening (Tian et al., 2007).

**FIGURE 3:**
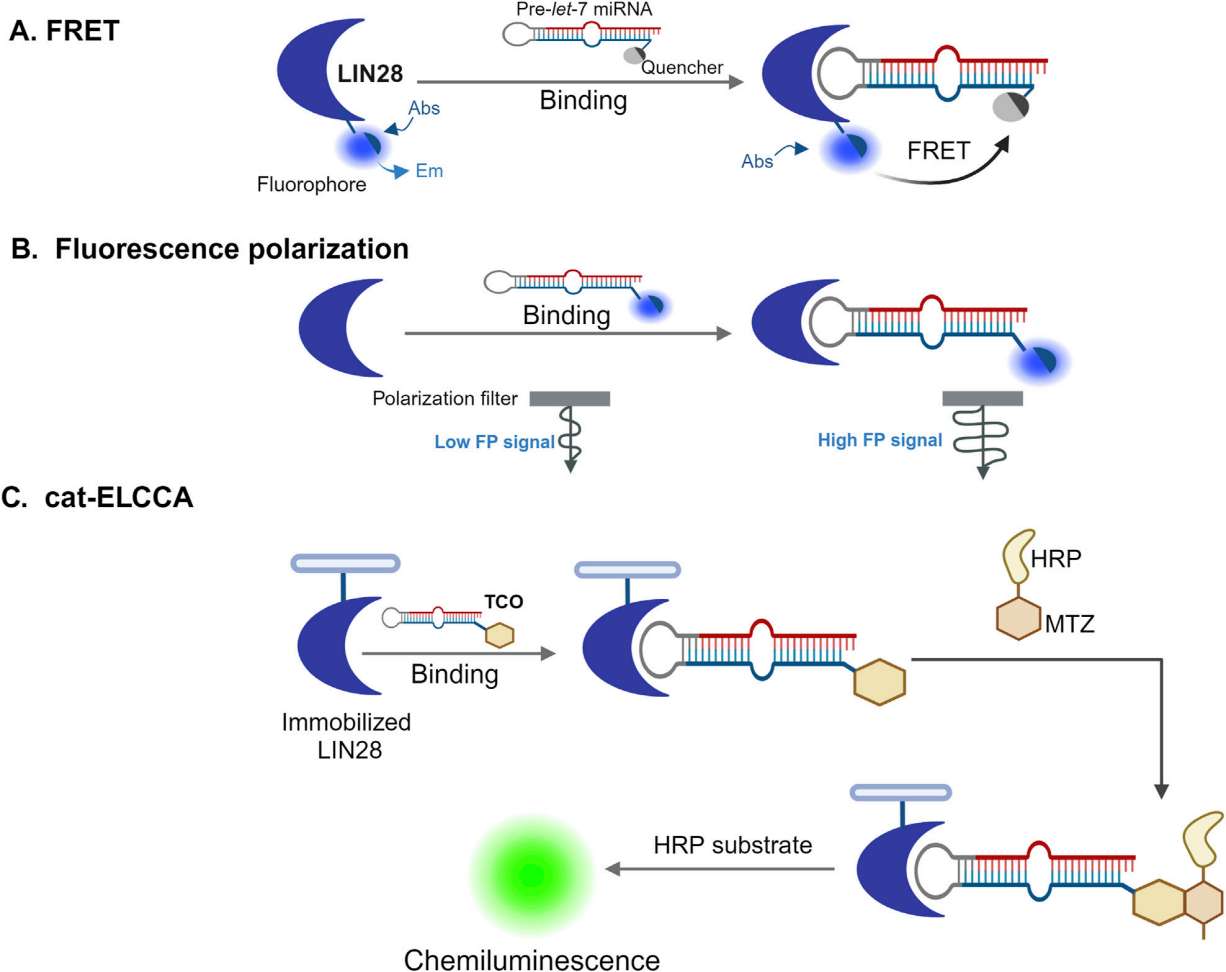
High-throughput screening approaches to identify RBPs inhibitors. Three different high-throughput screening strategies used for the identification of small-molecule inhibitors of RBPs **(A)** Fluorescence resonance energy transfer (FRET) assays (Lim et al., 2016). In this assay, a protein (in this example, LIN28) is labeled with a fluorophore that fluoresces stronger when it is on the free, unbound state. However, when the protein binds to a quencher-tagged precursor let-7 microRNA (pre-*let*-7 miRNA), the FRET between the fluorophore and the quencher leads to the reduction or elimination of fluorescence. Inhibitors that interfere with this interaction can prevent quenching, allowing the fluorescence to be detected. **(B)** Fluorescence polarization (FP) assay (Wang Longfei et al., 2018). In this assay, an RNA molecule is labeled with a fluorophore, which moves quickly when free and unbound, causing low emission of depolarized light on excitation with polarized light. However, when the RNA binds a protein, the rotation of the fluorophore is slowed down and the emission of polarized light is increased. If a compound interferes with the protein-RNA binding, the fluorophore can rotate more freely again, and the signal becomes depolarized, indicating a lower fluorescence polarization signal. **(C)** Catalytic enzyme-linked click-chemistry assay (cat-ELCCA) (Lorenz et al., 2018). In this assay, 5′-*trans*-cyclooctene (TCO)-labelled RNAs interact with immobilized LIN28 protein and their interaction is detected using a chemiluminescence signal. The signal arises from chemical reaction where the TCO-labeled RNA reacts with methyltetrazine (MTZ)-labeled horseradish peroxidase (HRP) followed by incubation with an HRP substrate. If a small molecule disrupts the RNA-LIN28 interaction, the chemiluminescence signal will decrease, indicating that the interaction has been weakened or blocked. Figure was created in BioRender. Bishola, T. (2025) https://BioRender.com/q33t189.

In contrast, newer fluorescence-based assays, such as Fluorescence Resonance Energy Transfer (FRET), are highly sensitive and capable of detecting the proximity of biomolecules, making them excellent tools for screening RNA-protein interactions. FRET works by labeling both a protein and its RNA target with different fluorescent probes, which allow detection of binding interactions based on changes in fluorescence signals (Janzen, 2014; Tian et al., 2007). Other fluorescence-based methods include Fluorescence Polarization Immunoassays (FPIA) and Fluorescence Lifetime Imaging Microscopy (FLIM) and fluorescence polarization assays (FP) (Smith and Eremin, 2008). FPIA is a user-friendly technique for monitoring small molecules, while FP assays are valuable for investigating protein-ligand binding affinities and thermo-stability (Huang et al., 2020). FLIM which exploits the lifetime property of fluorescence, is a microscopy technique that has gained popularity because of its high sensitivity to the molecular environment and changes in molecular conformation (Datta et al., 2020). Others assays including catalytic Enzyme-Linked Click Chemistry Assay (cat-ELCCA) as shown in Figure 3, are currently used along with native mass spectrometry (MS) (Garner, 2018). Although MS was initially used to study small proteins and protein−ligand interactions, the applications of native MS have expanded and cover a huge variety of protein assemblies involved in a plethora of different biological processes.

Recent studies have demonstrated the effectiveness of high-throughput screening (HTS) in identifying small molecules that disrupt RNA-binding protein (RBP) interactions. For example, a protein/RNA FRET assay using a GFP-tagged Lin28 donor and a Let-7 microRNA acceptor labeled with a black hole quencher (BHQ), as illustrated in Figure 3, enabled screening of over 16,000 small molecules. One compound, N-methyl-N-[3-(3-methyl[1,2,4]triazolo[4,3-b]pyridazin-6-yl)phenyl]acetamide, was identified as a disruptor of the Lin28/Let-7 interaction. This compound restored Let-7 processing and activity in cancer cells expressing Lin28, induced differentiation in mouse embryonic stem cells, and reduced tumor sphere formation in 22RV1 and Huh7 cancer cells. A biotinylated analog of the compound also successfully pulled down Lin28 from cell lysates, confirming target engagement within cells. The Lin28/Let-7 axis is an emerging therapeutic target in cancer biology, particularly for tumors that exploit Lin28 to repress tumor-suppressive Let-7 microRNAs (Julio and Backus, 2021; Roos et al., 2015; Roos et al., 2016; Zhang, 2008).

In another example, a fluorescence polarization (FP) assay as illustrated in Figure 3, optimized for HTS was used to screen a library of approximately 6,000 compounds for inhibitors of the HuR-ARE (AU-rich element) interaction. Several hits were validated using orthogonal methods, including AlphaLISA, surface plasmon resonance (SPR), ribonucleoprotein immunoprecipitation (RNP-IP), and luciferase reporter assays. These compounds disrupted HuR-RNA interactions at nanomolar concentrations and blocked HuR function by competitively binding to its RNA-binding site. Given HuR’s role in stabilizing oncogenic transcripts, these findings highlight the potential of these small molecules as chemical probes and possible leads for therapeutic development in cancers characterized by HuR overexpression (Wang et al., 2015).

FRET and HTS techniques have also led to the discovery of chemical probes targeting splicing-related RBPs, including Lin28, HuR, MSUT2, the ELF4 protein family, as well as other modulators such as cu-cpT17e, Branaplam, Risdiplam, RG7800, and splicing regulators like SMN-C1, -C2, -C3, and -C5. These discoveries have expanded the chemical biology toolkit for dissecting RBP function and hold therapeutic potential in diseases involving aberrant splicing and RNA regulation (Baker et al., 2020).

To select an appropriate assay, it is essential to consider the nature of the targets. For example, when an RNA-binding protein (RBP) is associated with spliceosome, a cell-based assay is recommended due to the complexity of the target. On the other hand, in a system containing only an RBP and an RNA molecule, a proximity-based fluorescent assay is more suitable for screening. It is crucial to define screening strategies tailored to a known target. Defining the right strategy is essential to minimize costs and avoid false positives. During the hit identification and lead discovery phase, screening assays are developed to identify compounds that show biologically relevant activity, allowing for the construction of a structure-activity relationship (SAR), which is critical for optimizing drug leads (Hughes et al., 2011). A “lead” is defined as a hit confirmed by more than one *assay in vitro*, and if possible *in vivo*, in a manner that shows biologically relevant activity that correlates to the target (Fox et al., 2006).

Fragment-based drug discovery also plays a crucial role in targeting protein-protein interactions (PPIs), which are notoriously challenging to modulate with small molecules. PPIs are often involved in key cellular processes, making their disruption or stabilization an important therapeutic strategy. FBDD approaches can identify compounds that either inhibit or stabilize PPIs by binding to one of the interacting proteins and affecting the protein-protein interaction.

Compounds targeting PPIs can achieve their effects by either disrupting interactions or stabilizing specific conformations to influence signaling pathways (Li, 2020). As drug discovery continues to evolve, screening strategies are becoming more integrated with advances in technology. HTS labs are increasingly using focused libraries, predictive profiling of compounds, and cherry-picking strategies to improve the efficiency and effectiveness of drug screening.

Systems biology and pharmacogenomics are gaining prominence as more screening strategies incorporate data-driven approaches. Moreover, HTS is now being applied in other areas, such as RNA interference research, biomarker discovery, and *in vitro* studies of absorption, distribution, metabolism, and excretion (ADME), as well as toxicity screening (Fox et al., 2006). The cost of screening remains a significant challenge for pharmaceutical companies, prompting many to outsource various stages of the process to reduce costs while improving the quality of leads generated through HTS.

Several promising drug leads have emerged from screening efforts aimed at treating tropical diseases such as the Human African Trypanosomiasis (HAT). Using FRET-based assays, researchers screened 100,000 compounds against purified *T. brucei* editosomes, identifying seven compounds that inhibited essential catalytic activities, such as RNA editing ligase activity (Rostamighadi et al., 2024). Virtual screening has also been employed to target specific enzymes involved in RNA editing, such as *Trypanosoma brucei* editing ligase 1 (TbREL1) (Salavati et al., 2012). Studies have identified compounds, such as GW 5074, mitoxantrone, and protoporphyrin IX, that inhibit RNA editing by interfering with endonuclease cleavage (Salavati et al., 2012). Other promising compounds, including fexinidazole, SCYX-7158, and acoziborole, have already entered clinical trials, highlighting the potential for high-throughput screening to identify effective treatments for neglected diseases like HAT (Gilbert, 2013).

### 5.2 Mechanisms of action of small molecules

**FIGURE 4:**
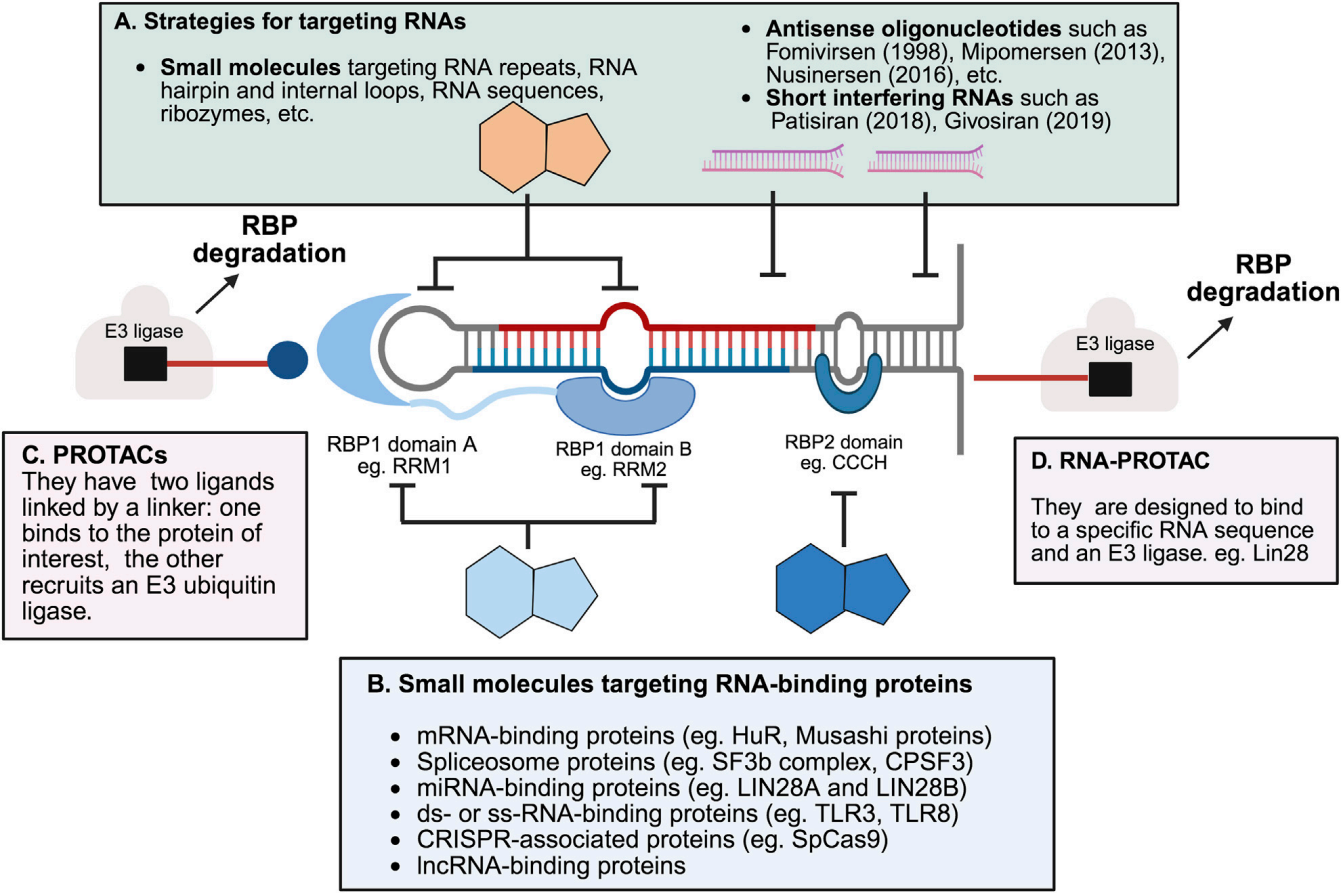
Selected strategies for targeting RNA-binding proteins and RNAs. **(A)** RNA-targeting strategies include nucleotide-based drugs such as fomivirsen (Crooke, 1998), mipomersen (Crooke et al., 2013), nusinersen (Scoto et al., 2018) and short interfering RNA (siRNA) such as patisiran (Setten et al., 2019) and givosiran (Sardh et al., 2019). These strategies are designed to target specific RNA sequences or structures (Angelbello et al., 2018). The year of regulatory approval of the drug by the US Food and Drug administration is indicated in parentheses. Small molecules targeting RNA can bind to structural motifs like hairpins and loops, RNA enzymes like ribozymes, or specific RNA sequences (such as those in HIV), as well as other RNA sequences and repeats. Some examples not shown include macrocyclic inhibitors and peptidomimetics (Shi et al., 2019). **(B)** Protein-targeting strategies involve small molecules and natural products that modulate RNA-binding proteins (RBPs) by interacting with their RNA-binding domains (D’Agostino et al., 2019). Protein-targeting small molecules modulate RNA-binding proteins (RBPs), which participate in diverse processes such as binding to mRNAs, microRNAs (miRNAs), double-stranded RNA (dsRNA), single-stranded RNA (ssRNA), and spliceosomal components, as well as other RNA-related functions, including those mediated by the bacterial CRISPR-Cas system. Other protein-targeting approaches, such as RNA constructs and antibody-based methods, are not covered here (Laver et al., 2012). **(C)** PROTACs (proteolysis-targeting chimeras) are heterobifunctional molecules that recruit E3 ubiquitin ligases to RNA-binding proteins, promoting their ubiquitination and subsequent proteasomal degradation. This strategy enables selective removal of disease-relevant RBPs rather than inhibition alone (Schreiber, 2021; Xiao et al., 2022). **(D)** RNA-PROTACs are chimeric structures that employ small RNA mimics as targeting groups to dock into the RNA-binding sites of RBPs. A conjugated E3-recruiting peptide, often derived from the HIF-1α protein, then directs the bound RBP to the ubiquitin–proteasome system for degradation (Ghidini et al., 2021; Modell et al., 2021). Figure was created in BioRender. Bishola, T. (2025) https://BioRender.com/htawgie.

Small molecules can influence the activity of RNA-binding proteins (RBPs) through several mechanisms (Julio and Backus, 2021) (as shown in Figure 4). One approach involves disrupting the interaction between RBPs and RNA. By preventing this binding, these compounds can alter RNA stability or affect signaling pathways (as illustrated in Figure 4). Such inhibitors are often identified using binding assays. The identified covalent inhibitors prevent RBPs from binding to RNA molecules. The formation of a covalent bond enhances the effectiveness and chemical characteristics of the modulators. Such inhibitors can be developed when an appropriate amino acid like serine, cysteine, or lysine is located within the binding site of the compound (Li and Kang, 2023).

Another strategy uses small molecules that improve the interactions between RNA molecule and protein or with components of the spliceosome. These types of compounds are usually found through biophysical assays. Molecules that bind directly to RNA can also influence how well RBPs can attach to it. In a different mechanism, small molecules binding to RBPs can trigger the degradation of RBPs by recruiting ubiquitin E3 ligases. The design of PROTACs (proteolysis-targeting chimeras) or molecular glues will result in modulators to reduce the level of RBPs (Corley et al., 2020; Wang L. et al., 2022).

Even though most known small molecule modulators interfere with RNA binding through direct competition or allosteric effect (see Figure 4), further chemical optimization is needed to meet the needs for clinical use. Interestingly, PROTACs can be developed by modifying existing RBP-binding compounds, without any inhibitory activity on RNA binding (as shown in Figure 4). Various screening methods, like fragment-based screening, molecular docking, or other binding assays, can be used to identify promising RBP-targeting molecules. Although RNA molecules were once considered as undruggable targets, recent research shows that small molecules can selectively bind RNAs (Datta et al., 2020; Huang et al., 2020; Smith and Eremin, 2008; Tian et al., 2007). RNA binding to an RBP can also result in conformational changes, which might create new binding sites for drug development (Flores and Ataide, 2018). Therefore, it might be possible to design compounds that stabilize RBP-RNA complexes as a way to treat certain diseases.

### 5.3 Bifunctional molecules

Proteolysis-targeting chimeras (PROTACs) are a class of bifunctional small molecules designed to induce the targeted degradation of proteins by harnessing the cell’s ubiquitin-proteasome system (Zhong J. et al., 2024). They consist of three components: a ligand that binds the protein of interest (POI), a ligand that recruits an E3 ubiquitin ligase, and a linker that connects these two ligands (Pasieka et al., 2023). Despite their potential, the clinical application of PROTACs faces challenges, including issues with specificity, pharmacokinetics, pharmacodynamics, and large-scale production. These limitations have driven the development of heterobifunctional molecules, which can simultaneously interact with two or more targets to induce the desired molecular interactions or reactions. Recent advancements have led to the design of several types of heterobifunctional molecules. These include traditional PROTACs which bind to both an RBP and an E3 ligase, facilitating the degradation of the RBP through the ubiquitin-proteasome pathway; RNA-PROTACs which are designed to bind to a specific RNA sequence and an E3 ligase (as illustrated in Figure 4). When a small molecule that binds to the RNA sequence is available, linking this molecule with an E3 ligase-binding moiety can result in a compound that affects the RBP level through induced ubiquitination (Li and Kang, 2023). RIBOTACs (Ribonuclease-Targeting Chimeras) are another type of heterobifunctional molecules which induce RNA degradation by binding to both RNA and a ribonuclease, such as RNase L. Linking small molecule binders of RBPs with an RNase-binding moiety can result in a molecule that degrades RNA by recruiting the RNase to proximity with the RNA molecule (Modell et al., 2021). Lastly, bifunctional molecules targeting both RBPs and RNA bind to both an RBP and an RNA sequence, potentially improving the specificity of individual moieties and offering more precise modulation of RBP function (Li and Kang, 2023).

The development of these heterobifunctional molecules provides new opportunities for drug discovery and development, particularly in targeting RNA-binding proteins (RBPs) that have been challenging to modulate with traditional small molecules. However, designing such compounds remains complex due to the chemical properties and structural intricacies of RBP–RNA complexes. Ongoing research is essential to overcome these challenges and realize the therapeutic potential of these innovative molecules.

## 6 Challenges in clinical translation of RBPs targeting therapeutics

Despite significant progress in targeting RNA-binding proteins (RBPs) through modalities such as natural products, PROTACs, and covalent inhibitors, translating these approaches into clinical therapies faces significant modality-specific barriers. Natural products often present synthetic challenges due to their complex structures, which complicates scale-up, while their promiscuous binding can lead to off-target effects and toxicity. PROTACs, meanwhile, are hindered by their high molecular weight (typically over 700 Da), which adversely affects cell permeability, oral bioavailability, and tissue distribution. The design of the linker joining the target ligand and E3 ligase is particularly critical to activity, as linker length, flexibility, and rigidity directly influence ternary complex formation, target selectivity, and pharmacokinetic behavior (Cecchini et al., 2021). Macrocyclization or introduction of rigid scaffolds in linker design can improve potency, specificity, and cellular efficacy even if binary binding affinity to the target is reduced (Li and Crews, 2023). Additionally, strategies such as PEGylation, nanoparticle delivery, and antibody-conjugated PROTACs are being investigated to address pharmacokinetic and targeting challenges. AI-assisted modeling tools that predict ternary complex stability and linker behavior are increasingly applied to rationally optimize PROTACs as drug-like entities (Fu et al., 2025; Lin et al., 2025; Lv et al., 2025).

Covalent inhibitors pose their own hurdles: electrophilic warheads may modify nucleophilic residues across the proteome, raising concerns around off-target toxicity. Strategies to mitigate these risks include the development of “soft” electrophiles with tuned reactivity and the application of chemo-proteomic profiling methods (e.g., ABPP, residue-based probes) to identify and eliminate unintended targets (Wai Cheung et al., 2021). Reversible covalent inhibitor designs also offer promise in balancing sustained target engagement with improved safety margins (Zhao and Bourne, 2025).

Finally, to bridge the gap from mechanistic studies to clinical application, a cohesive framework that links structural modulation of RBP domains to downstream transcriptomic and phenotypic changes is essential. Multi-omics integration, including RNA sequencing, proteomics, and chemo-proteomics, helps trace the impact of RBP perturbation across molecular networks, while biomarker-based selection (such as RBP expression signatures or splicing variants) can enable precision targeting. Predictive modeling that combines AI-driven ADME-Tox evaluation with protein–ligand docking further accelerates rational optimization and clinical de-risking.

## 7 Conclusion and future prospects

Targeting RNA-binding proteins (RBPs) with small-molecule inhibitors is a growing field of research, offering a complementary approach to modulate protein-RNA interactions. Over the past decade, significant progress has been made in understanding RBP functions and identifying small-molecule modulators, many of which were once considered “undruggable” (Julio and Backus, 2021; Li and Kang, 2023). High-throughput screening has been key in discovering these inhibitors, often followed by structure-activity relationship (SAR) analysis to refine compounds. Notable progress has been made with RBPs like LIN28 (Rosenblum et al., 2024), RNA-sensing toll-like receptors (Zheng et al., 2023), and Cas proteins (Chen et al., 2022), but challenges remain in developing more effective inhibitors with better biological and pharmacological properties. As more RBP structures are solved, the drug discovery process is expected to shift from screening to rational drug design (Julio and Backus, 2021). Techniques like NMR and computational modeling are playing a crucial role in identifying potential inhibitors by leveraging structural information from related proteins. The diversity of chemical libraries is also critical for discovering compounds with novel biological effects. Libraries containing both protein- and RNA-targeting molecules are needed to explore protein-RNA interactions fully.

In addition to RNA binding, RBPs may have other functions, such as DNA binding, pseudokinase activity, or protein-protein interactions, offering new avenues for targeting RBP functions (Corley et al., 2020). These non-RNA-binding activities could offer new opportunities for targeting RBP functions in different ways. Moreover, proteins that interact with RBPs without directly binding to RNA, such as some recently identified RNA-dependent proteins, could provide an alternative approach for modulating protein–RNA interactions, especially when small molecules that directly target either the protein or RNA are not available. While significant strides have been made in identifying small-molecule inhibitors for a variety of RBPs, further research is required to better understand their mechanisms of action, confirm their efficacy in different assays, and evaluate their performance in living organisms. With continued development, RBP inhibitors hold potential for chemical biology and biomedical applications, offering novel treatments for diseases such as trypanosomiasis, cancer, autoimmune and inflammatory disorders, and neurodegenerative diseases.

## References

[B1] Agam G. Gebhardt C. Popara M. Mächtel R. Folz J. Ambrose B. (2023). Reliability and accuracy of single-molecule FRET studies for characterization of structural dynamics and distances in proteins. Nat. Methods 20 (4), 523–535. 10.1038/s41592-023-01807-0 36973549 PMC10089922

[B2] Ali M. A. Caetano-Anollés G. (2024). AlphaFold2 reveals structural patterns of seasonal haplotype diversification in SARS-CoV-2 nucleocapsid protein variants. Viruses 16 (9), 1358. 10.3390/v16091358 39339835 PMC11435742

[B3] Angelbello A. J. Chen J. L. Childs-Disney J. L. Zhang P. Wang Z.-F. Disney M. D. (2018). Using genome sequence to enable the design of medicines and chemical probes. Chem. Rev. 118 (4), 1599–1663. 10.1021/acs.chemrev.7b00504 29322778 PMC5989578

[B4] Antoine C. Allain F. H.-T. (2012). “From structure to function of RNA binding domains,” in RNA bind proteins. Editor Lorkovic Z. J. (Boca Raton, FL: Landes Bioscience), 137–158.

[B5] Araki S. Ohori M. Yugami M. (2023). Targeting pre-mRNA splicing in cancers: roles, inhibitors, and therapeutic opportunities. Front. Oncol. 13 (June), 1152087–16. 10.3389/fonc.2023.1152087 37342192 PMC10277747

[B6] Baker J. D. Uhrich R. L. Strovas T. J. Saxton A. D. Kraemer B. C. (2020). Targeting Pathological Tau by small molecule inhibition of the poly(A):MSUT2 RNA-protein interaction. ACS Chem. Neurosci. 11 (15), 2277–2285. 10.1021/acschemneuro.0c00214 32589834 PMC8629322

[B7] Barnwal R. P. Yang F. Varani G. (2017). Applications of NMR to structure determination of RNAs large and small. Arch. Biochem. Biophys. 628 (1), 42–56. 10.1016/j.abb.2017.06.003 28600200 PMC5555312

[B8] Bayona J. C. Nakayasu E. S. Laverrière M. Aguilar C. Sobreira T. J. P. Choi H. (2011). SUMOylation pathway in Trypanosoma cruzi: functional characterization and proteomic analysis of target proteins. Mol. Cell Proteomics 10 (12), M110.007369–13. 10.1074/mcp.M110.007369 21832256 PMC3237068

[B9] Begolo D. Vincent I. M. Giordani F. Pöhner I. Witty M. J. Rowan T. G. (2018). The trypanocidal benzoxaborole AN7973 inhibits trypanosome mRNA processing. PLoS Pathog. 14, e1007315–e1007333. 10.1371/journal.ppat.1007315 30252911 PMC6173450

[B10] Békés M. Langley D. R. Crews C. M. (2022). PROTAC targeted protein degraders: the past is prologue. Nat. Rev. Drug Discov. 21 (3), 181–200. 10.1038/s41573-021-00371-6 35042991 PMC8765495

[B11] Betu Kumeso V. K. Kalonji W. M. Rembry S. Valverde Mordt O. Ngolo Tete D. Prêtre A. (2023). Efficacy and safety of acoziborole in patients with human African trypanosomiasis caused by Trypanosoma brucei gambiense: a multicentre, open-label, single-arm, phase 2/3 trial. Lancet Infect. Dis. 23 (4), 463–470. 10.1016/s1473-3099(22)00660-0 36460027 PMC10033454

[B12] Bheemireddy S. Sandhya S. Srinivasan N. Sowdhamini R. (2022). Computational tools to study RNA-protein complexes. Front. Mol. Biosci. 9 (October), 954926–17. 10.3389/fmolb.2022.954926 36275618 PMC9585174

[B13] Bishola T. T. Clayton C. (2022). Interactions of the Trypanosoma brucei brucei zinc-finger-domain protein ZC3H28. Parasitology 149 (3), 356–370. 10.1017/s003118202100189x 35264260 PMC10090614

[B14] Blanco F. F. Preet R. Aguado A. Vishwakarma V. Stevens L. E. Vyas A. (2016). Impact of HuR inhibition by the small molecule MS-444 on colorectal cancer cell tumorigenesis. Oncotarget 7 (45), 74043–74058. 10.18632/oncotarget.12189 27677075 PMC5342034

[B15] Booth T. J. Kalaitzis J. A. Vuong D. Crombie A. Lacey E. Piggott A. M. (2020). Production of novel pladienolide analogues through native expression of a pathway-specific activator. Chem. Sci. 11 (31), 8249–8255. 10.1039/d0sc01928c 34094178 PMC8163091

[B16] Brennan C. M. Steitz J. A. (2001). HuR and mRNA stability. Cell Mol. Life Sci. 58 (2), 266–277. 10.1007/PL00000854 11289308 PMC11146503

[B17] Cantara W. A. Olson E. D. Musier-Forsyth K. (2017). Analysis of RNA structure using small-angle X-ray scattering. Methods. 113, 46–55. 10.1016/j.ymeth.2016.10.008 27777026 PMC5253320

[B18] Cassandri M. Smirnov A. Novelli F. Pitolli C. Agostini M. Malewicz M. (2017). Zinc-finger proteins in health and disease. Cell Death Discov. 3 (1), 17071. 10.1038/cddiscovery.2017.71 29152378 PMC5683310

[B19] Castello A. Fischer B. Frese C. K. Horos R. Alleaume A.-M. Foehr S. (2016). Comprehensive identification of RNA-binding domains in human cells. Mol. Cell. 63 (4), 696–710. 10.1016/j.molcel.2016.06.029 27453046 PMC5003815

[B20] Cecchini C. Pannilunghi S. Tardy S. Scapozza L. (2021). From conception to development: investigating PROTACs features for improved cell permeability and successful protein degradation. Front. Chem. 9 (April), 672267–23. 10.3389/fchem.2021.672267 33959589 PMC8093871

[B21] Chaudhari R. Fong L. W. Tan Z. Huang B. Zhang S. (2020). An up-to-date overview of computational polypharmacology in modern drug discovery. Expert Opin. Drug Discov. 15 (9), 1025–1044. 10.1080/17460441.2020.1767063 32452701 PMC7415563

[B22] Chen X. Kopecky D. J. Mihalic J. Jeffries S. Min X. Heath J. (2012). Structure-guided design, synthesis, and evaluation of guanine-derived inhibitors of the eIF4E mRNA–cap interaction. J. Med. Chem. 55 (8), 3837–3851. 10.1021/jm300037x 22458568

[B23] Chen S. Chen D. Liu B. Haisma H. J. (2022). Modulating CRISPR/Cas9 genome-editing activity by small molecules. Drug Discov. Today 27 (4), 951–966. 10.1016/j.drudis.2021.11.018 34823004

[B24] Chen Y. Xia Z. Suwal U. Rappu P. Heino J. De Wever O. (2024). Dual-ligand PROTACS mediate superior target protein degradation *in vitro* and therapeutic efficacy *in vivo* . Chem. Sci. 15 (42), 17691–17701. 10.1039/d4sc03555k 39391379 PMC11462456

[B25] Cheung A. K. Hurley B. Kerrigan R. Shu L. Chin D. N. Shen Y. (2018). Discovery of small molecule splicing modulators of survival motor neuron-2 (SMN2) for the treatment of spinal muscular atrophy (SMA). J. Med. Chem. 61 (24), 11021–11036. 10.1021/acs.jmedchem.8b01291 30407821

[B26] Clayton C. (2013). The regulation of trypanosome gene expression by RNA-binding proteins. PLoS Pathog. 9 (11), e1003680–12. 10.1371/journal.ppat.1003680 24244152 PMC3820711

[B27] Clayton C. (2019). Regulation of gene expression in trypanosomatids: living with polycistronic transcription. Open Biol. 9 (6), 190072. 10.1098/rsob.190072 31164043 PMC6597758

[B28] Corley M. Burns M. C. Yeo G. W. (2020). How RNA-binding proteins interact with RNA: molecules and mechanisms. Mol. Cell 78 (1), 9–29. 10.1016/j.molcel.2020.03.011 32243832 PMC7202378

[B29] Crooke S. T. (1998). Vitravene--another piece in the mosaic. Antisense Nucleic Acid. Drug Dev. 8. 10.1089/oli.1.1998.8.vii 9743463

[B30] Crooke S. T. Geary R. S. (2013). Clinical pharmacological properties of mipomersen (Kynamro), a second generation antisense inhibitor of apolipoprotein B. Br. J. Clin. Pharmacol. 76 (2), 269–276. 10.1111/j.1365-2125.2012.04469.x 23013161 PMC3731601

[B31] Daina A. Michielin O. Zoete V. (2017). SwissADME: a free web tool to evaluate pharmacokinetics, drug-likeness and medicinal chemistry friendliness of small molecules. Sci. Rep. 7 (October 2016), 42717–13. 10.1038/srep42717 28256516 PMC5335600

[B32] Dalpke A. H. Helm M. (2012). RNA mediated Toll-like receptor stimulation in health and disease. RNA Biol. 9 (6), 828–842. 10.4161/rna.20206 22617878 PMC3495747

[B33] Datta R. Heaster T. M. Sharick J. T. Gillette A. A. Skala M. C. (2020). Fluorescence lifetime imaging microscopy: fundamentals and advances in instrumentation, analysis, and applications. J. Biomed. Opt. 25 (07), 1–44. 10.1117/1.jbo.25.7.071203 32406215 PMC7219965

[B34] De Vries T. Martelly W. Campagne S. Sabath K. Sarnowski C. P. Wong J. (2022). Sequence-specific RNA recognition by an RGG motif connects U1 and U2 snRNP for spliceosome assembly. Proc. Natl. Acad. Sci. U. S. A. 119 (6), e2114092119–11. 10.1073/pnas.2114092119 35101980 PMC8833184

[B35] Dey S. K. Jaffrey S. R. (2019). RIBOTACs: small molecules target RNA for degradation. Cell Chem. Biol. 26 (8), 1047–1049. 10.1016/j.chembiol.2019.07.015 31419417

[B36] Du X. Volkov O. A. Czerwinski R. M. Tan H. L. Huerta C. Morton E. R. (2019). Structural basis and kinetic pathway of RBM39 recruitment to DCAF15 by a sulfonamide molecular glue E7820. Structure 27 (11), 1625–1633.e3. 10.1016/j.str.2019.10.005 31693911

[B37] D’Agostino V. G. Sighel D. Zucal C. Bonomo I. Micaelli M. Lolli G. (2019). Screening approaches for targeting ribonucleoprotein complexes: a new dimension for drug discovery. SLAS Discov. 24 (3), 314–331. 10.1177/2472555218818065 30616427

[B38] Fang Y. Liu X. Liu Y. Xu N. (2024). Insights into the mode and mechanism of interactions between RNA and RNA-binding proteins. Int. J. Mol. Sci. 25 (21), 11337. 10.3390/ijms252111337 39518890 PMC11545484

[B39] Flores J. K. Ataide S. F. (2018). Structural changes of RNA in complex with proteins in the SRP. Front. Mol. Biosci. 5 (FEB), 7–8. 10.3389/fmolb.2018.00007 29459899 PMC5807370

[B40] Fox S. Farr-Jones S. Sopchak L. Boggs A. Nicely H. W. Khoury R. (2006). High-throughput screening: update on practices and success. J. Biomol. Screen 11 (7), 864–869. 10.1177/1087057106292473 16973922

[B41] Fu S. Zhu X. Huang F. Chen X. (2025). Anti-PEG antibodies and their biological impact on PEGylated drugs: challenges and strategies for optimization. Pharmaceutics 17 (8), 1074–22. 10.3390/pharmaceutics17081074 40871093 PMC12388889

[B42] Fukuda T. Kawakami K. Toyoda M. Hayashi C. Sanui T. Uchiumi T. (2024). Luteolin, chemical feature and potential use for oral disease. Curr. Oral Heal Rep. 11, 290–296. 10.1007/s40496-024-00389-w

[B43] Garner A. L. (2018). Cat-ELCCA: catalyzing drug discovery through click chemistry. Chem. Commun. 50 (54), 6531–6539. 10.1039/c8cc02332h 29781014 PMC6008226

[B44] Ge J. Li S. Weng G. Wang H. Fang M. Sun H. (2025). PROTAC-DB 3.0: an updated database of PROTACs with extended pharmacokinetic parameters. Nucleic Acids Res. 53 (D1), D1510–D1515. 10.1093/nar/gkae768 39225044 PMC11701630

[B45] Gerstberger S. Hafner M. Tuschl T. (2014). A census of human RNA-binding proteins. Nat. Rev. Genet. 15 (12), 829–845. 10.1038/nrg3813 25365966 PMC11148870

[B46] Geuens T. Bouhy D. Timmerman V. (2016). The hnRNP family: insights into their role in health and disease. Hum. Genet. 135 (8), 851–867. 10.1007/s00439-016-1683-5 27215579 PMC4947485

[B47] Ghidini A. Cléry A. Halloy F. Allain F. H. T. Hall J. (2021). RNA-PROTACs: degraders of RNA-binding proteins. Angew. Chem. - Int. Ed. 60 (6), 3163–3169. 10.1002/anie.202012330 33108679 PMC7898822

[B48] Gilbert I. H. (2013). Drug discovery for neglected diseases: molecular target-based and phenotypic approaches. J. Med. Chem. 56 (20), 7719–7726. 10.1021/jm400362b 24015767 PMC3954685

[B49] Gollner A. Rudolph D. Arnhof H. Bauer M. Blake S. M. Boehmelt G. (2016). Discovery of novel spiro[3H-indole-3,2’-pyrrolidin]-2(1H)-one compounds as chemically stable and orally active inhibitors of the MDM2-p53 interaction. J. Med. Chem. 59 (22), 10147–10162. 10.1021/acs.jmedchem.6b00900 27775892

[B50] Grishin N. V. (2001). KH domain: one motif, two folds. Nucleic Acids Res. 29 (3), 638–643. 10.1093/nar/29.3.638 11160884 PMC30387

[B51] Gutnik D. Evseev P. Miroshnikov K. Shneider M. (2023). Using AlphaFold predictions in viral research. Curr. Issues Mol. Biol. 45 (4), 3705–3732. 10.3390/cimb45040240 37185764 PMC10136805

[B52] Han T. Goralski M. Gaskill N. Capota E. Kim J. Ting T. C. (2017). Anticancer sulfonamides target splicing by inducing RBM39 degradation via recruitment to DCAF15. Science. 356 (6336), eaal3755. 10.1126/science.aal3755 28302793

[B53] Haniff H. S. Tong Y. Liu X. Chen J. L. Suresh B. M. Andrews R. J. (2020). Targeting the SARS-COV-2 RNA genome with small molecule binders and ribonuclease targeting chimera (RiboTAC) degraders. ACS Cent. Sci. 6 (10), 1713–1721. 10.1021/acscentsci.0c00984 33140033 PMC7553039

[B54] Harvey A. L. Edrada-Ebel R. Quinn R. J. (2015). The re-emergence of natural products for drug discovery in the genomics era. Nat. Rev. Drug Discov. 14 (2), 111–129. 10.1038/nrd4510 25614221

[B55] He S. Valkov E. Cheloufi S. Murn J. (2023). The nexus between RNA-binding proteins and their effectors. Nat. Rev. Genet. 24 (5), 276–294. 10.1038/s41576-022-00550-0 36418462 PMC10714665

[B56] Heinemann U. Roske Y. (2021). Cold-shock domains—abundance, structure, properties, and nucleic-acid binding. Cancers (Basel) 13 (2), 190–22. 10.3390/cancers13020190 33430354 PMC7825780

[B57] Huang X. Tang X. Jallow A. Qi X. Zhang W. Jiang J. (2020). Development of an ultrasensitive and rapid fluorescence polarization immunoassay for ochratoxin A in rice. Toxins (Basel). 12 (11), 682. 10.3390/toxins12110682 33138019 PMC7693749

[B58] Huang X. Wu F. Ye J. Wang L. Wang X. Li X. (2024). Expanding the horizons of targeted protein degradation: a non-small molecule perspective. Acta Pharm. Sin. B 14 (6), 2402–2427. 10.1016/j.apsb.2024.01.010 38828146 PMC11143490

[B59] Hughes J. P. Rees S. S. Kalindjian S. B. Philpott K. L. (2011). Principles of early drug discovery. Br. J. Pharmacol. 162 (6), 1239–1249. 10.1111/j.1476-5381.2010.01127.x 21091654 PMC3058157

[B60] Igoli N. P. Obanu Z. A. Gray A. I. Clements C. (2012). Bioactive diterpenes and sesquiterpenes from the rhizomes of wild ginger (Siphonochilus aethiopicus (Schweinf) B.L Burtt). Afr. J. Tradit. Complement. Altern. Med. 9 (1), 88–93. 10.4314/ajtcam.v9i1.13 23983325 PMC3746532

[B61] Jaiswal A. K. Thaxton M. L. Scherer G. M. Sorrentino J. P. Garg N. K. Rao D. S. (2024). Small molecule inhibition of RNA binding proteins in haematologic cancer. RNA Biol. 21 (1), 276–289. 10.1080/15476286.2024.2303558 38329136 PMC10857685

[B62] Janzen W. P. (2014). Screening technologies for small molecule discovery: the state of the art. Chem. Biol. 21 (9), 1162–1170. 10.1016/j.chembiol.2014.07.015 25237860

[B63] Järvelin A. I. Noerenberg M. Davis I. Castello A. (2016). The new (dis)order in RNA regulation. Cell Commun. Signal 14 (1), 9. 10.1186/s12964-016-0132-3 27048167 PMC4822317

[B64] Jia Y. Oyken M. Kim R. Q. Tjokrodirijo R. T. N. de Ru A. H. Janssen A. P. A. (2024). Development of inhibitors, probes, and PROTAC provides a complete toolbox to study PARK7 in the living cell. J. Med. Chem. 67 (10), 7935–7953. 10.1021/acs.jmedchem.3c02410 38713163 PMC11129182

[B65] Julio A. R. Backus K. M. (2021). New approaches to target RNA binding proteins. Curr. Opin. Chem. Biol. 62, 13–23. 10.1016/j.cbpa.2020.12.006 33535093 PMC8823266

[B66] Jungfleisch J. Gebauer F. (2025). RNA-binding proteins as therapeutic targets in cancer. cancer. RNA Biol. 22 (1), 1–8. 10.1080/15476286.2025.2470511 40016176 PMC11869776

[B67] Kaida D. Motoyoshi H. Tashiro E. Nojima T. Hagiwara M. Ishigami K. (2007). Spliceostatin A targets SF3b and inhibits both splicing and nuclear retention of pre-mRNA. Nat. Chem. Biol. 3 (9), 576–583. 10.1038/nchembio.2007.18 17643111

[B68] Kiely-Collins H. Winter G. E. Bernardes G. J. L. (2021). The role of reversible and irreversible covalent chemistry in targeted protein degradation. Cell Chem. Biol. 28 (7), 952–968. 10.1016/j.chembiol.2021.03.005 33789091

[B69] Kilim O. Mentes A. Pál B. Csabai I. Gellért Á. (2023). SARS-CoV-2 receptor-binding domain deep mutational AlphaFold2 structures. Sci. Data 10 (1), 134–138. 10.1038/s41597-023-02035-z 36918581 PMC10013278

[B70] Kotake Y. Sagane K. Owa T. Mimori-Kiyosue Y. Shimizu H. Uesugi M. (2007). Splicing factor SF3b as a target of the antitumor natural product pladienolide. Nat. Chem. Biol. 3 (9), 570–575. 10.1038/nchembio.2007.16 17643112

[B71] Lagisetti C. Pourpak A. Jiang Q. Cui X. Goronga T. Morris S. W. (2008). Antitumor compounds based on a natural product consensus pharmacophore. J. Med. Chem. 51 (19), 6220–6224. 10.1021/jm8006195 18788726 PMC2701350

[B72] Lambrecht L. Arnold P. Behr J. Mertsch P. Tufman A. Kauffmann-Guerrero D. (2024). Topotecan in a real-world small-cell lung cancer cohort: prognostic biomarkers improve selection of patients for second-line treatment. Diagnostics 14 (14), 1572–12. 10.3390/diagnostics14141572 39061709 PMC11276225

[B73] Laver J. D. Ancevicius K. Sollazzo P. Westwood J. T. Sidhu S. S. Lipshitz H. D. (2012). Synthetic antibodies as tools to probe RNA-binding protein function. Mol. Biosyst. 8 (6), 1650–1657. 10.1039/C2MB00007E 22481296

[B74] Lei X. Zheng Y. Su W. (2025). RNA-binding proteins and autophagy in lung cancer: mechanistic insights and therapeutic perspectives. Discov. Oncol. 16 (1), 599. 10.1007/s12672-025-02413-6 40272614 PMC12022210

[B75] Li Q. (2020). Application of fragment-based drug discovery to versatile targets. Front. Mol. Biosci. 7 (August), 180–13. 10.3389/fmolb.2020.00180 32850968 PMC7419598

[B76] Li K. Crews C. M. (2023). PROTACs: past, present and future. Chem. Soc. Rev. 51 (12), 5214–5236. 10.1039/d2cs00193d 35671157 PMC10237031

[B77] Li S.-J. Hochstrasser M. (2000). The yeast ULP2 (SMT4) gene encodes a novel protease specific for the ubiquitin-like Smt3 protein. Mol. Cell Biol. 20 (7), 2367–2377. 10.1128/mcb.20.7.2367-2377.2000 10713161 PMC85410

[B78] Li Q. Kang C. (2023). Targeting RNA-binding proteins with small molecules: perspectives, pitfalls and bifunctional molecules. FEBS Lett. 597 (16), 2031–2047. 10.1002/1873-3468.14710 37519019

[B79] Li Z. Guo Q. Zhang J. Fu Z. Wang Y. Wang T. (2021a). The RNA-binding motif protein family in cancer: friend or foe? Front. Oncol. 11 (November), 757135–15. 10.3389/fonc.2021.757135 34804951 PMC8600070

[B80] Li X. Pu W. Chen S. Peng Y. (2021b). Therapeutic targeting of RNA-binding protein by RNA-PROTAC. Mol. Ther. 29 (6), 1940–1942. 10.1016/j.ymthe.2021.04.032 33984279 PMC8178457

[B81] Li Y. Jia Y. Wang X. Shang H. Tian Y. (2022). Protein-targeted degradation agents based on natural products. Pharm. (Basel) 16 (1), 46. 10.3390/ph16010046 36678543 PMC9865760

[B82] Li J. Chen X. Lu A. Liang C. (2023). Targeted protein degradation in cancers: orthodox PROTACs and beyond. Innovation 4 (3), 100413. 10.1016/j.xinn.2023.100413 37033156 PMC10074253

[B83] Li P. Hu X. Fan Z. Sun S. Ran Q. Wei T. (2024). Novel potent molecular glue degraders against broad range of hematological cancer cell lines via multiple neosubstrates degradation. J. Hematol. Oncol. 17 (1), 77. 10.1186/s13045-024-01592-z 39218923 PMC11367868

[B84] Lim D. Byun W. G. Koo J. Y. Park H. Park S. B. (2016). Discovery of a small-molecule inhibitor of protein–MicroRNA interaction using binding assay with a site-specifically labeled Lin28. J. Am. Chem. Soc. 138 (41), 13630–13638. 10.1021/jacs.6b06965 27668966

[B85] Lin J. Zhou D. Steitz T. A. Polikanov Y. S. Gagnon M. G. (2018). Ribosome-targeting antibiotics: modes of action, mechanisms of resistance, and implications for drug design. Annu. Rev. Biochem. 87, 451–478. 10.1146/annurev-biochem-062917-011942 29570352 PMC9176271

[B86] Lin J. Chen Z. Zhang D. Zhang N. Chen H. Guo D.-S. (2025). Integrating proteolysis-targeting chimeras (PROTACs) with delivery systems for more efficient and precise targeted protein degradation. Macromol. Rapid Commun. 46 (14), e2401051. 10.1002/marc.202401051 40183416

[B87] Lindquist J. A. Mertens P. R. (2018). Cold shock proteins: from cellular mechanisms to pathophysiology and disease. Cell Commun. Signal 16 (1), 63–14. 10.1186/s12964-018-0274-6 30257675 PMC6158828

[B88] Ling S. C. Albuquerque C. P. Han J. S. Lagier-Tourenne C. Tokunaga S. Zhou H. (2010). ALS-associated mutations in TDP-43 increase its stability and promote TDP-43 complexes with FUS/TLS. Proc. Natl. Acad. Sci. U. S. A. 107 (30), 13318–13323. 10.1073/pnas.1008227107 20624952 PMC2922163

[B89] Lorenz D. A. Kaur T. Kerk S. A. Gallagher E. E. Sandoval J. Garner A. L. (2018). Expansion of cat-ELCCA for the discovery of small molecule inhibitors of the pre-let-7-lin28 RNA-protein interaction. ACS Med. Chem. Lett. 9 (6), 517–521. 10.1021/acsmedchemlett.8b00126 29937975 PMC6004563

[B90] Loughlin F. E. Wilce J. A. (2019). TDP-43 and FUS–structural insights into RNA recognition and self-association. Curr. Opin. Struct. Biol. 59, 134–142. 10.1016/j.sbi.2019.07.012 31479821

[B91] Lovci M. T. Bengtson M. H. Massirer K. B. (2016). Post-translational modifications and RNA-binding proteins. Adv. Exp. Med. Biol. 907, 297–317. 10.1007/978-3-319-29073-7_12 27256391

[B92] Lunde B. M. Moore C. Varani G. (2007). RNA-binding proteins: modular design for efficient function. Nat. Rev. Mol. Cell Biol. 8 (6), 479–490. 10.1038/nrm2178 17473849 PMC5507177

[B93] Lv W. Jia X. Tang B. Ma C. Fang X. Jin X. (2025). *In silico* modeling of targeted protein degradation. Eur. J. Med. Chem. 289, 117432. 10.1016/j.ejmech.2025.117432 40015161

[B94] Macarron R. Banks M. N. Bojanic D. Burns D. J. Cirovic D. A. Garyantes T. (2011). Impact of high-throughput screening in biomedical research. Nat. Rev. Drug Discov. 10 (3), 188–195. 10.1038/nrd3368 21358738

[B95] Maris C. Dominguez C. Allain F. H. T. (2005). The RNA recognition motif, a plastic RNA-binding platform to regulate post-transcriptional gene expression. FEBS J. 272 (9), 2118–2131. 10.1111/j.1742-4658.2005.04653.x 15853797

[B96] Masliah G. Barraud P. Allain F. H. T. (2013). RNA recognition by double-stranded RNA binding domains: a matter of shape and sequence. Cell Mol. Life Sci. 70 (11), 1875–1895. 10.1007/s00018-012-1119-x 22918483 PMC3724394

[B97] Masuzawa T. Oyoshi T. (2020). Roles of the RGG domain and RNA recognition motif of nucleolin in G-quadruplex stabilization. ACS Omega 5 (10), 5202–5208. 10.1021/acsomega.9b04221 32201808 PMC7081427

[B98] Mayr F. Schütz A. Döge N. Heinemann U. (2012). The Lin28 cold-shock domain remodels pre-let-7 microRNA. Nucleic Acids Res. 40 (15), 7492–7506. 10.1093/nar/gks355 22570413 PMC3424542

[B99] Meisner N. C. Hintersteiner M. Mueller K. Bauer R. Seifert J. M. Naegeli H. U. (2007). Identification and mechanistic characterization of low-molecular-weight inhibitors for HuR. Nat. Chem. Biol. 3 (8), 508–515. 10.1038/nchembio.2007.14 17632515

[B100] Mizui Y. Sakai T. Iwata M. Uenaka T. Okamoto K. Shimizu H. (2004). Pladienolides, new substances from culture of Streptomyces platensis Mer-11107. III. *in vitro* and *in vivo* antitumor activities. J. Antibiot. (Tokyo) 57 (3), 188–196. 10.7164/antibiotics.57.188 15152804

[B101] Modell A. E. Lai S. Nguyen T. M. Choudhary A. (2021). Bifunctional modalities for repurposing protein function. Cell Chem. Biol. 28 (7), 1081–1089. 10.1016/j.chembiol.2021.06.005 34270935

[B102] Mowbray C. E. Braillard S. Glossop P. A. Whitlock G. A. Jacobs R. T. Speake J. (2021). DNDI-6148: a novel benzoxaborole preclinical candidate for the treatment of visceral leishmaniasis. J. Med. Chem. 64 (21), 16159–16176. 10.1021/acs.jmedchem.1c01437 34711050 PMC8591608

[B103] Muppirala U. K. Honavar V. G. Dobbs D. (2011). Predicting RNA-protein interactions using only sequence information. BMC Bioinforma. 12 (1), 489. 10.1186/1471-2105-12-489 22192482 PMC3322362

[B104] Nag S. Qin J. Srivenugopal K. S. Wang M. Zhang R. (2013). The MDM2-p53 pathway revisited. J. Biomed. Res. 27 (4), 254–271. 10.7555/jbr.27.20130030 23885265 PMC3721034

[B105] Nagasawa R. Onizuka K. Komatsu K. R. Miyashita E. Murase H. Ojima K. (2024). Large-scale analysis of small molecule-RNA interactions using multiplexed RNA structure libraries. Commun. Chem. 7 (1), 98–12. 10.1038/s42004-024-01181-8 38693284 PMC11865577

[B106] Naryshkin N. A. Weetall M. Dakka A. Narasimhan J. Zhao X. Feng Z. (2014). *SMN2* splicing modifiers improve motor function and longevity in mice with spinal muscular atrophy. Science. 345 (6197), 688–693. 10.1126/science.1250127 25104390

[B107] Newman D. J. Cragg G. M. (2020). Natural products as sources of new drugs over the nearly four decades from 01/1981 to 09/2019. J. Nat. Prod. 83 (3), 770–803. 10.1021/acs.jnatprod.9b01285 32162523

[B108] Nguyen T. C. Zaleta-Rivera K. Huang X. Dai X. Zhong S. (2018). RNA, action through interactions. Trends Genet. 34 (11), 867–882. 10.1016/j.tig.2018.08.001 30177410 PMC6195460

[B109] Ogunleye O. O. Jatau I. D. Natala A. J. Ola-Fadunsin S. D. (2020). Effects of aqueous extract of fruit pulp of Adansonia digitata L. on the oxidative stress profile against Trypanosoma brucei brucei infection in albino rats. Clin. Phytoscience 6 (1), 57. 10.1186/s40816-020-00203-x

[B110] Olejniczak M. Jiang X. Basczok M. M. Storz G. (2022). KH domain proteins: another family of bacterial RNA matchmakers? Mol. Microbiol. 117 (1), 10–19. 10.1111/mmi.14842 34748246 PMC8766902

[B111] Owa T. Yoshino H. Okauchi T. Yoshimatsu K. Ozawa Y. Sugi N. H. (1999). Discovery of novel antitumor sulfonamides targeting G1 phase of the cell cycle. J. Med. Chem. 42 (19), 3789–3799. 10.1021/jm9902638 10508428

[B112] Palacino J. Swalley S. E. Song C. Cheung A. K. Shu L. Zhang X. (2015). SMN2 splice modulators enhance U1–pre-mRNA association and rescue SMA mice. Nat. Chem. Biol. 11 (7), 511–517. 10.1038/nchembio.1837 26030728

[B113] Pan X. Rijnbeek P. Yan J. Shen H. B. (2018). Prediction of RNA-protein sequence and structure binding preferences using deep convolutional and recurrent neural networks. BMC Genomics 19 (1), 511–11. 10.1186/s12864-018-4889-1 29970003 PMC6029131

[B114] Pasieka A. Diamanti E. Uliassi E. Laura Bolognesi M. (2023). Click chemistry and targeted degradation: a winning combination for medicinal chemists? ChemMedChem 18 (20), e202300422. 10.1002/cmdc.202300422 37706617 PMC13652707

[B115] Pereira B. Billaud M. Almeida R. (2017). RNA-binding proteins in cancer: old players and new actors. Trends cancer 3 (7), 506–528. 10.1016/j.trecan.2017.05.003 28718405

[B116] Qin H. Ni H. Liu Y. Yuan Y. Xi T. Li X. (2020). RNA-binding proteins in tumor progression. J. Hematol. Oncol. 13 (1), 90–23. 10.1186/s13045-020-00927-w 32653017 PMC7353687

[B117] Qiu C. Dutcher R. C. Porter D. F. Arava Y. Wickens M. Hall T. M. T. (2019). Distinct RNA-binding modules in a single PUF protein cooperate to determine RNA specificity. Nucleic Acids Res. 47 (16), 8770–8784. 10.1093/nar/gkz583 31294800 PMC7145691

[B118] Qiu J. Wu L. Qu R. Jiang T. Bai J. Sheng L. (2022). History of development of the life-saving drug “Nusinersen” in spinal muscular atrophy. Front. Cell Neurosci. 16 (August), 942976–14. 10.3389/fncel.2022.942976 36035257 PMC9414009

[B119] Raisinghani N. Alshahrani M. Gupta G. Xiao S. Tao P. Verkhivker G. (2024). Exploring conformational landscapes and binding mechanisms of convergent evolution for the SARS-CoV-2 spike Omicron variant complexes with the ACE2 receptor using AlphaFold2-based structural ensembles and molecular dynamics simulations. Phys. Chem. Chem. Phys. 26 (25), 17720–17744. 10.1039/d4cp01372g 38869513

[B120] Ratni H. Karp G. M. Weetall M. Naryshkin N. A. Paushkin S. V. Chen K. S. (2016). Specific correction of alternative survival motor neuron 2 splicing by small molecules: discovery of a potential novel medicine to treat spinal muscular atrophy. J. Med. Chem. 59 (13), 6086–6100. 10.1021/acs.jmedchem.6b00459 27299419

[B121] Ratni H. Ebeling M. Baird J. Bendels S. Bylund J. Chen K. S. (2018). Discovery of risdiplam, a selective survival of motor neuron-2 (SMN2) gene splicing modifier for the treatment of spinal muscular atrophy (SMA). J. Med. Chem. 61 (15), 6501–6517. 10.1021/acs.jmedchem.8b00741 30044619

[B122] Rhodes C. Balaratnam S. Yazdani K. Seshadri S. Schneekloth J. S. (2024). Targeting RNA-protein interactions with small molecules: promise and therapeutic potential. Med. Chem. Res. 33 (11), 2050–2065. 10.1007/s00044-024-03342-9

[B123] Romano M. Buratti E. (2013). Targeting RNA binding proteins involved in neurodegeneration. J. Biomol. Screen 18 (9), 967–983. 10.1177/1087057113497256 23954928

[B124] Roos M. Rebhan M. A. E. Lucic M. Pavlicek D. Pradere U. Towbin H. (2015). Short loop-targeting oligoribonucleotides antagonize Lin28 and enable pre-let-7 processing and suppression of cell growth in let-7-deficient cancer cells. Nucleic Acids Res. 43 (2), e9. 10.1093/nar/gku1090 25378324 PMC4333367

[B125] Roos M. Pradère U. Ngondo R. P. Behera A. Allegrini S. Civenni G. (2016). A small-molecule inhibitor of Lin28. ACS Chem. Biol. 11 (10), 2773–2781. 10.1021/acschembio.6b00232 27548809

[B126] Rosenblum S. L. Soueid D. M. Giambasu G. Vander Roest S. Pasternak A. DiMauro E. F. (2024). Live cell screening to identify RNA-binding small molecule inhibitors of the pre-let-7-Lin28 RNA-protein interaction. RSC Med. Chem. 15 (5), 1539–1546. 10.1039/d4md00123k 38784453 PMC11110735

[B127] Rostamighadi M. Kamelshahroudi A. Mehta V. Zeng F. Y. Pass I. Chung T. D. Y. (2024). High-throughput screening of compounds targeting RNA editing in Trypanosoma brucei: novel molecular scaffolds with broad trypanocidal effects. Biochem. Pharmacol. 219 (November 2023), 115937. 10.1016/j.bcp.2023.115937 37995979

[B128] Salavati R. Moshiri H. Kala S. Shateri Najafabadi H. (2012). Inhibitors of RNA editing as potential chemotherapeutics against trypanosomatid pathogens. Int. J. Parasitol. Drugs Drug Resist 2, 36–46. 10.1016/j.ijpddr.2011.10.003 24533263 PMC3862403

[B129] Salicioni A. M. Xi M. Vanderveer L. A. Balsara B. Testa J. R. Dunbrack R. L. J. (2000). Identification and structural analysis of human RBM8A and RBM8B: two highly conserved RNA-binding motif proteins that interact with OVCA1, a candidate tumor suppressor. Genomics 69 (1), 54–62. 10.1006/geno.2000.6315 11013075

[B130] Sardh E. Harper P. Balwani M. Stein P. Rees D. Bissell D. M. (2019). Phase 1 trial of an RNA interference therapy for acute intermittent porphyria. N. Engl. J. Med. 380 (6), 549–558. 10.1056/nejmoa1807838 30726693

[B131] Sasso J. M. Tenchov R. Wang D. S. Johnson L. S. Wang X. Zhou Q. A. (2023). Molecular glues: the adhesive connecting targeted protein degradation to the clinic. Biochemistry 62 (3), 601–623. 10.1021/acs.biochem.2c00245 35856839 PMC9910052

[B132] Schmeing S. Hart P. T. (2024). Challenges in therapeutically targeting the RNA-recognition motif. Wiley Interdiscip. Rev. RNA 15 (6), e1877. 10.1002/wrna.1877 39668490 PMC11638515

[B133] Schreiber S. L. (2021). The rise of molecular glues. Cell 184 (1), 3–9. 10.1016/j.cell.2020.12.020 33417864

[B134] Scoto M. Finkel R. Mercuri E. Muntoni F. (2018). Genetic therapies for inherited neuromuscular disorders. Lancet Child. Adolesc. Heal 2 (8), 600–609. 10.1016/s2352-4642(18)30140-8 30119719

[B135] Seiler M. Yoshimi A. Darman R. Chan B. Keaney G. Thomas M. (2018). H3B-8800, an orally available small-molecule splicing modulator, induces lethality in spliceosome-mutant cancers. Nat. Med. 24 (4), 497–504. 10.1038/nm.4493 29457796 PMC6730556

[B136] Setten R. L. Rossi J. J. Han S. (2019). The current state and future directions of RNAi-based therapeutics. Nat. Rev. Drug Discov. 18 (6), 421–446. 10.1038/s41573-019-0017-4 30846871

[B137] Shi Y. Parag S. Patel R. Lui A. Murr M. Cai J. (2019). Stabilization of lncRNA GAS5 by a small molecule and its implications in diabetic adipocytes. Cell Chem. Biol. 26 (3), 319–330.e6. 10.1016/j.chembiol.2018.11.012 30661991 PMC10498384

[B138] Shi Y. Bray W. Smith A. J. Zhou W. Calaoagan J. Lagisetti C. (2020). An exon skipping screen identifies antitumor drugs that are potent modulators of premRNA splicing, suggesting new therapeutic applications. PLoS One 15 (5), e0233672–19. 10.1371/journal.pone.0233672 32469945 PMC7259758

[B139] Sievers Q. L. Petzold G. Bunker R. D. Renneville A. Słabicki M. Liddicoat B. J. (2018). Defining the human C2H2 zinc finger degrome targeted by thalidomide analogs through CRBN. Sci. 362 (6414), eaat0572. 10.1126/science.aat0572 30385546 PMC6326779

[B140] Singh N. K. Singh N. N. Androphy E. J. Singh R. N. (2006). Splicing of a critical exon of human survival motor neuron is regulated by a unique silencer element located in the last intron. Mol. Cell Biol. 26 (4), 1333–1346. 10.1128/mcb.26.4.1333-1346.2006 16449646 PMC1367187

[B141] Singh R. N. Ottesen E. W. Singh N. N. (2020). The first orally deliverable small molecule for the treatment of spinal muscular atrophy. Neurosci. Insights 15, 2633105520973985. 10.1177/2633105520973985 33283185 PMC7691903

[B142] Sivaramakrishnan M. McCarthy K. D. Campagne S. Huber S. Meier S. Augustin A. (2017). Binding to SMN2 pre-mRNA-protein complex elicits specificity for small molecule splicing modifiers. Nat. Commun. 8 (1), 1476. 10.1038/s41467-017-01559-4 29133793 PMC5684323

[B143] Smith D. S. Eremin S. A. (2008). Fluorescence polarization immunoassays and related methods for simple, high-throughput screening of small molecules. Anal. Bioanal. Chem. 391 (5), 1499–1507. 10.1007/s00216-008-1897-z 18264817

[B144] Soomro Z. Youssef M. Yust-Katz S. Jalali A. Patel A. J. Mandel J. (2020). Paraneoplastic syndromes in small cell lung cancer. J. Thorac. Dis. 12 (10), 6253–6263. 10.21037/jtd.2020.03.88 33209464 PMC7656388

[B145] Spinello A. Borišek J. Malcovati L. Magistrato A. (2021). Investigating the molecular mechanism of H3B-8800: a splicing modulator inducing preferential lethality in spliceosome-mutant cancers. Int. J. Mol. Sci. 22 (20), 11222. 10.3390/ijms222011222 34681880 PMC8540225

[B146] Sternburg E. L. Karginov F. V. (2020). Global approaches in studying RNA-binding protein interaction networks. Trends Biochem. Sci. 45 (7), 593–603. 10.1016/j.tibs.2020.03.005 32531229

[B147] Tants J. N. Schlundt A. (2023). Advances, applications, and perspectives in small-angle X-ray scattering of RNA. ChemBioChem. 24 (17), e202300110. 10.1002/cbic.202300110 37466350

[B148] Thompson R. D. Baisden J. T. Zhang Q. (2019). NMR characterization of RNA small molecule interactions. Physiol. Behav. 176 (5), 139–148. 10.1016/j.ymeth.2019.05.015 31128236 PMC6756962

[B149] Tian B. Bevilacqua P. C. Diegelman-Parente A. Mathews M. B. (2004). The double-stranded-RNA-binding motif: interference and much more. Nat. Rev. Mol. Cell Biol. 5 (12), 1013–1023. 10.1038/nrm1528 15573138

[B150] Tian H. Ip L. Luo H. Chang D. C. Luo K. Q. (2007). A high throughput drug screen based on fluorescence resonance energy transfer (FRET) for anticancer activity of compounds from herbal medicine. Br. J. Pharmacol. 150 (3), 321–334. 10.1038/sj.bjp.0706988 17179946 PMC2013898

[B151] Valverde R. Edwards L. Regan L. (2008). Structure and function of KH domains. FEBS J. 275 (11), 2712–2726. 10.1111/j.1742-4658.2008.06411.x 18422648

[B152] Varadi M. Bertoni D. Magana P. Paramval U. Pidruchna I. Radhakrishnan M. (2024). AlphaFold Protein Structure Database in 2024: providing structure coverage for over 214 million protein sequences. Nucleic Acids Res. 52 (November 2023), D368–D375. 10.1093/nar/gkad1011 37933859 PMC10767828

[B153] Vasilyev N. Polonskaia A. Darnell J. C. Darnell R. B. Patel D. J. Serganov A. (2015). Crystal structure reveals specific recognition of a G-quadruplex RNA by a β-turn in the RGG motif of FMRP. Proc. Natl. Acad. Sci. U. S. A. 112 (39), E5391–E5400. 10.1073/pnas.1515737112 26374839 PMC4593078

[B154] Wai Cheung C. Sharifzadeh S. Buhrlage S. J. Marto J. A. (2021). Chemoproteomic methods for covalent drug discovery. Chem. Soc. Rev. 50 (15), 8361–8381. 10.1039/d1cs00231g 34143170 PMC8328943

[B155] Waithaka A. Clayton C. (2022). Clinically relevant benzoxaboroles inhibit mRNA processing in Trypanosoma brucei. BMC Res. Notes 15 (1), 371–376. 10.1186/s13104-022-06258-y 36528767 PMC9758897

[B156] Wall R. J. Rico E. Lukac I. Zuccotto F. Elg S. Gilbert I. H. (2018). Clinical and veterinary trypanocidal benzoxaboroles target CPSF3. Proc. Natl. Acad. Sci. U. S. A. 115 (38), 9616–9621. 10.1073/pnas.1807915115 30185555 PMC6156652

[B157] Walters K. Sajek M. P. Murphy E. Issaian A. Baldwin A. Harrison E. (2023). Small-molecule Ro-08-2750 interacts with many RNA-binding proteins and elicits MUSASHI2-independent phenotypes. Rna 29 (10), 1458–1470. 10.1261/rna.079605.123 37369529 PMC10578479

[B158] Wan X. Yang T. Cuesta A. Pang X. Balius T. E. Irwin J. (2020). Discovery of lysine-targeted eIF4E inhibitors through covalent docking. J. Am. Chem. Soc. 142 (11), 4960–4964. 10.1021/jacs.9b10377 32105459 PMC7136196

[B159] Wang X. Zamore P. D. Hall T. M. (2001). Crystal structure of a Pumilio homology domain. Mol. Cell 7 (4), 855–865. 10.1016/s1097-2765(01)00229-5 11336708

[B160] Wang Z. Bhattacharya A. Ivanov D. N. (2015). Identification of Small-Molecule Inhibitors of the HuR/RNA Interaction using a fluorescence polarization screening assay followed by NMR validation. PLoS One 10 (9), e0138780. 10.1371/journal.pone.0138780 26390015 PMC4577092

[B161] Wang M. Ogé L. Perez-Garcia M. D. Hamama L. Sakr S. (2018a). The PUF protein family: overview on PUF RNA targets, biological functions, and post transcriptional regulation. Int. J. Mol. Sci. 19 (2), 1–13. 10.3390/ijms19020410 29385744 PMC5855632

[B162] Wang L. Rowe R. G. Jaimes A. Yu C. Nam Y. Pearson D. S. (2018b). Small-molecule inhibitors disrupt let-7 oligouridylation and release the selective blockade of let-7 processing by LIN28. Cell Rep. 23 (10), 3091–3101. 10.1016/j.celrep.2018.04.116 29874593 PMC6511231

[B163] Wang J. Hjelmeland A. B. Nabors L. B. King P. H. (2019). Anti-cancer effects of the HuR inhibitor, MS-444, in malignant glioma cells. Cancer Biol. Ther. 20 (7), 979–988. 10.1080/15384047.2019.1591673 30991885 PMC6606032

[B164] Wang S. Sun Z. Lei Z. Zhang H.-T. (2022a). RNA-binding proteins and cancer metastasis. Semin. Cancer Biol. 86, 748–768. 10.1016/j.semcancer.2022.03.018 35339667

[B165] Wang L. Gao J. Ma R. Liu Y. Liu M. Zhong F. (2022b). Recent progress in fragment-based drug discovery facilitated by NMR spectroscopy. Magn. Reson Lett. 2 (2), 107–118. 10.1016/j.mrl.2021.100025

[B166] Wang Y. Zhang J. Deng J. Wang C. Fang L. Zhang Y. (2023). Targeted degradation of DNA/RNA binding proteins via covalent hydrophobic tagging. CCS Chem. 5 (10), 2207–2214. 10.31635/ccschem.023.202302873

[B167] Wang X. Li J. Zhang C. Guan X. Li X. Jia W. (2025). Old players and new insights: unraveling the role of RNA-binding proteins in brain tumors. Theranostics 15 (11), 5238–5257. 10.7150/thno.113312 40303323 PMC12036871

[B168] Wheeler E. C. Martin B. J. E. Doyle W. C. Neaher S. Conway C. A. Pitton C. N. (2024). Splicing modulators impair DNA damage response and induce killing of cohesin-mutant MDS and AML. Sci. Transl. Med. 16 (728), eade2774. 10.1126/scitranslmed.ade2774 38170787 PMC11222919

[B169] Wu X. Xu L. (2022). The RNA-binding protein HuR in human cancer: a friend or foe? Adv. Drug Deliv. Rev. 184, 114179–26. 10.1016/j.addr.2022.114179 35248670 PMC9035123

[B170] Wu X. Gardashova G. Lan L. Han S. Zhong C. Marquez R. T. (2020). Targeting the interaction between RNA-binding protein HuR and FOXQ1 suppresses breast cancer invasion and metastasis. Commun. Biol. 3 (1), 193. 10.1038/s42003-020-0933-1 32332873 PMC7181695

[B171] Xiao M. Zhao J. Wang Q. Liu J. Ma L. (2022). Recent advances of degradation technologies based on PROTAC mechanism. Biomolecules 12 (9), 1257–15. 10.3390/biom12091257 36139095 PMC9496103

[B172] Xu X. Vatsyayan J. Gao C. Bakkenist C. J. Hu J. (2010). Sumoylation of eIF4E activates mRNA translation. EMBO Rep. 11 (4), 299–304. 10.1038/embor.2010.18 20224576 PMC2854592

[B173] Xue G. Chen J. Liu L. Zhou D. Zuo Y. Fu T. (2020). Protein degradation through covalent inhibitor-based PROTACs. Chem. Commun. 56 (10), 1521–1524. 10.1039/c9cc08238g 31922153

[B174] Yoshimoto R. Chhipi-Shrestha J. K. Schneider-Poetsch T. Furuno M. Burroughs A. M. Noma S. (2021). Spliceostatin A interaction with SF3B limits U1 snRNP availability and causes premature cleavage and polyadenylation. Cell Chem. Biol. 28 (9), 1356–1365.e4. 10.1016/j.chembiol.2021.03.002 33784500

[B175] Zanoteli E. França M. C. Marques W. (2024). Gene-based therapies for neuromuscular disorders. Arq. Neuropsiquiatr. 82 (6), 001–010. 10.1055/s-0043-1777755 38325390 PMC10849828

[B176] Zhang X. D. (2008). Novel analytic criteria and effective plate designs for quality control in genome-scale RNAi screens. J. Biomol. Screen 13 (5), 363–377. 10.1177/1087057108317062 18567841

[B177] Zhang J. Ferré-D’Amar A. R. (2014). New molecular engineering approaches for crystallographic studies of large RNAs. Curr. Opin. Struct. Biol. 0 (1), 9–15. 10.1016/j.sbi.2014.02.001 PMC4127099 24607443

[B178] Zhao Z. Bourne P. E. (2025). Advances in reversible covalent kinase inhibitors. Med. Res. Rev. 45 (2), 629–653. 10.1002/med.22084 39287197 PMC11796325

[B179] Zhao X. Chang F. Lv H. Zou G. Zhang B. (2023). A novel deep learning method for predicting RNA-protein binding sites. Appl. Sci. 13 (5), 3247. 10.3390/app13053247

[B180] Zheng X. Peng Q. Wang L. Zhang X. Huang L. Wang J. (2020). Serine/arginine-rich splicing factors: the bridge linking alternative splicing and cancer. Int. J. Biol. Sci. 16 (13), 2442–2453. 10.7150/ijbs.46751 32760211 PMC7378643

[B181] Zheng H. Wu P. Bonnet P. A. (2023). Recent advances on small-molecule antagonists targeting TLR7. Molecules 28 (2), 634–19. 10.3390/molecules28020634 36677692 PMC9865772

[B182] Zhong G. Chang X. Xie W. Zhou X. (2024a). Targeted protein degradation: advances in drug discovery and clinical practice. Signal Transduct. Target Ther. 9 (1), 308. 10.1038/s41392-024-02004-x 39500878 PMC11539257

[B183] Zhong J. Zhao R. Wang Y. Su Y. X. Lan X. (2024b). Nano-PROTACs: state of the art and perspectives. Nanoscale 16 (9), 4378–4391. 10.1039/d3nr06059d 38305466

[B184] Zhou Y. Xiao Y. (2020). Chemoproteomic-driven discovery of covalent PROTACs. Biochemistry 59 (2), 128–129. 10.1021/acs.biochem.9b00795 31538467

[B185] Zigdon I. Carmi M. Brodsky S. Rosenwaser Z. Barkai N. Jonas F. (2024). Beyond RNA-binding domains: determinants of protein-RNA binding. RNA 30 (12), 1620–1633. 10.1261/rna.080026.124 39353735 PMC11571813

